# Multimodal predictors of spoken vocabulary development in autism: the role of early childhood brain and behavior

**DOI:** 10.1038/s41398-026-04168-2

**Published:** 2026-06-19

**Authors:** Olivia Surgent, Letitia Naigles, Andrew Dakopolos, Val K. Sierakowski, Derek S. Andrews, Joshua K. Lee, Brianna Heath, Sally J. Rogers, David G. Amaral, Marjorie Solomon, Christine Wu Nordahl

**Affiliations:** 1 MIND Institute and Department of Psychiatry and Behavioral Sciences, UC Davis School of Medicine, University of California Davis, Sacramento, CA USA; 2 Department of Psychological Sciences, University of Connecticut, Storrs, CT USA

**Keywords:** Neuroscience, Predictive markers, Human behaviour, Autism spectrum disorders

## Abstract

Many autistic children have spoken vocabulary delays that impact communication abilities during critical developmental periods. Behavioral and brain predictors of spoken language development in autism have been independently identified but rarely evaluated simultaneously. Here, we examined how fine motor skills, joint attention, and white matter microstructure of speech-related tracts are associated with spoken vocabulary development in autistic children with a broad range of speech abilities. Spoken vocabulary was assessed longitudinally in 122 autistic children (38% female) first between 2–4 years (Time 1) and again approximately two years later. Children were divided into groups based on their Time 1 spoken vocabularies: larger, average, or smaller. Fifty-eight autistic children (47% of the sample) were in the smaller vocabulary group, which represented both emergent speakers and non-speaking individuals. Within this smaller vocabulary group, faster rates of spoken vocabulary development were associated with better Time 1 fine motor and joint attention skills, and lower Time 1 fractional anisotropy (FA) in the occipital inferior longitudinal fasciculus, central arcuate fasciculus, and inferior corticospinal tract. Rates of spoken vocabulary development were best predicted by models that included both brain and behavior metrics, which each individually explained significant variance and together accounted for roughly half of the model variability. Results suggest that together early childhood fine motor skills, joint attention skills, and white matter microstructure contribute to a developmental cascade supporting spoken language. Findings demonstrate the potential clinical utility of early brain scanning, indicating that microstructure is predictive of language development above and beyond salient behavioral predictors.

## Introduction

For many children, the development of a robust spoken vocabulary unlocks new opportunities to communicate with the human world. However, 30% to 65% of young autistic children have delays in developing spoken language that impact their ability to speak to others during this critical period of early life [1]. While some autistic toddlers with early speech delays never develop complex spoken language, others experience rapid development over the childhood years, resulting in age-appropriate spoken vocabularies by elementary school [1–6]. Distinct behavioral and neurobiological correlates of concurrent speech and predictors of future spoken language outcomes have been identified in subsets of the autistic population [7–9]. However, it remains unclear how early childhood brain and behavioral factors together account for the variability in spoken vocabulary development observed across young autistic children, especially those with speech delays. Identifying a combination of early biological and behavioral predictors of spoken vocabulary development may be particularly relevant for 1) identifying autistic children at the highest likelihood to remain minimally speaking throughout childhood and 2) supporting the individual needs of all autistic children with diverse trajectories of spoken language development [10]. Therefore, the purpose of the present study was to examine whether measures of early childhood behavior and brain structure in combination predict spoken vocabulary development more accurately than either measure alone, using a longitudinal cohort of autistic children.

Several behavioral, familial, and environmental factors have been identified as predictors of speech development in autistic children [4, 11–13]. Fine motor (FM) and joint attention (JA) skills are two particularly salient predictors as they are both early developing skills that can be assessed in a clinical context and are directly associated with spoken language outcomes in autistic children who are minimally speaking at young ages [2, 7, 9, 11–18]. Specifically, FM skills, which involve precise control of the hands and fingers, and JA skills, which involve the ability to initiate coordinated attention with others, may contribute to a larger spoken language developmental cascade by providing foundational tools that promote vocabulary learning. Strong FM skills may reflect greater oral-motor proficiency resulting in fluent speech and may facilitate spoken language development by promoting direct engagement with items that can be talked about [19]. JA skills contribute to early spoken language development by enabling children to successfully direct a caregiver’s attention to stimuli of interest and to associate the caregiver’s spoken response with a specific item or event [13, 14]. Through these mechanisms, both FM and JA skills serve as essential early building blocks for later development of spoken language, making them strong candidates for prognostic evaluation and individualized early intervention approaches. Yet, few studies have investigated how FM and JA skills work together to contribute to speech outcomes in autistic individuals [2, 16] and no studies have investigated whether these behaviorally-based predictions of spoken language development can be strengthened by neurobiological measures.

In the brain, spoken language development emerges from the coordinated maturation of distributed white matter networks that support perception, integration, and production of speech. White matter pathways such as the inferior longitudinal fasciculus (ILF), arcuate fasciculus (AF), uncinate fasciculus (UF), and corticospinal tract (CST) form components of these broader neural systems that contribute to language learning and speech [20–22]. Together, these pathways structurally link brain regions involved in audiovisual and linguistic integration, semantic and socio-communicative processing, and motor control of articulatory musculature. Differences in each of these tracts have been observed in autistic individuals, suggesting that neurobiological variance may contribute to differences in spoken language development in this population [23, 24]. Yet, few studies have examined neural correlates of spoken language development in minimally speaking autistic children, in part due to perceived MRI acquisition challenges in this population [25]. To mitigate these challenges, our group developed techniques for collecting MRI data from young autistic children during natural (non-sedated) nocturnal sleep [25] and demonstrated that occipital ILF microstructure differed in minimally speaking autistic children compared to speaking peers [8]. Additionally, studies of minimally speaking autistic and non-autistic youth have identified concurrent microstructural differences in portions of the UF, AF, and CST compared to speaking peers [26, 27], providing corroborating evidence that differences in these white matter tracts may constrain or facilitate spoken language development. Yet, to our knowledge, no studies have tested whether early white matter differences in these pathways can add to behaviorally-based predictions of later spoken language outcomes in autism, nor have they tested whether neurobiological metrics explain variance beyond behavioral predictors of spoken language development; if so, such information could strengthen conceptual and therapeutic approaches to supporting speech development in autism.

Therefore, the goal of the present study was to predict spoken vocabulary development in young autistic children using measures of both behavior (JA and FM skills) and white matter microstructure. Spoken vocabulary levels were assessed longitudinally, once between two and four years of age (Time 1) and again roughly two years later (Follow-up), representing a key window for spoken language development. Based on Time 1 spoken vocabulary levels, children were categorized into one of three groups: “smaller starting spoken vocabulary” (spoken vocabulary levels below a typically developing two-year-old), “average starting spoken vocabulary” (spoken vocabulary levels consistent with a typically developing three-year-old, the average chronological age of the sample at Time 1), and “larger starting spoken vocabulary” (spoken vocabulary levels above a typically developing four-year-old). Importantly, children in the smaller starting spoken vocabulary group represented emergent or minimally speaking individuals, a population at elevated risk for persistent communication limitations and for whom identifying early predictors of spoken language development is especially critical. Accordingly, analyses were designed to first characterize predictors of spoken vocabulary development within this vulnerable group and to then test the specificity and generalizability of effects across the broader autistic sample. Based on previous work examining predictors of language development in minimally speaking autistic children, we hypothesized that better Time 1 FM and JA skills and specific patterns of Time 1 white matter microstructural properties (higher fractional anisotropy [FA] and axial diffusivity [AD], lower mean diffusivity [MD] and radial diffusivity [RD]), in the ILF, AF, UF, and CST would be independently associated with higher rates of spoken vocabulary learning over time, especially in the smaller starting vocabulary group [2, 8, 15, 16]. We also hypothesized that early differences in Time 1 microstructure and behavior combined would best predict spoken vocabulary development in the smaller starting vocabulary group.

## Methods

### Participants

122 autistic participants enrolled in the UC Davis MIND Institute’s Autism Phenome Project (APP) or Girls with Autism Imaging of Neurodevelopment (GAIN) Study; the latter was initiated to increase female representation in the broader sample [28]. Study protocols largely overlap, and the cohorts are combined. Behavioral and MRI data from study enrollment (Time 1) at approximately three years of age and behavioral data from a follow-up at approximately five years of age (Follow-up/Time 3) were utilized. “Time 2” visits from the APP/GAIN studies did not include behavioral assessments and therefore were not used in the current analyses. Previous language analysis at Time 1 has been conducted in an all-male subset of the sample [8]. All participants were English-speaking and not diagnosed with severe motor, vision, hearing or chronic health complications that could impair participation.

At Time 1, diagnostic confirmation of autism was conducted by a licensed clinical psychologist using 1) the Autism Diagnostic Observation Schedule (ADOS)-Generic [29] or ADOS-2 [30] and 2) the Autism Diagnostic Interview-Revised [31]. At Follow-up, diagnostic status was assessed using the ADOS-2. Table 1 contains additional participant information.

**Table 1:** Demographics.

	All	Starting Spoken Vocabulary Subgroups				
		Larger	Average	Smaller		
Time 1	(*n* = 122)	(*n* = 39)	(*n* = 25)	(*n* = 58)	F	*p*
Age (Months)	36.6(5.4)	39.2(4.6)	37.1(5.6)	34.8(5.08)	8.09	<0.001
Mean (SD), Range	24.6-50.0	27.4-49.2	24.8-50.1	24.6-45.4		
Sex (n Female [%])	45(37.8%)	21(17.2%)	16(13.1%)	8(6.56%)	6.67^a^	0.04
EOWPVT raw score	17.5(16.5)	37.6(10.4)	19.7(3.4)	3.2(4.2)	291.4	<0.001
Mean (SD), Range	0–74	26–74	14–25	0–12		
Joint Attention (ADOS-2)	6.6(2.0)	5.4(1.4)	6.8(1.7)	7.4(2.1)	13.38	<0.001
Mean (SD), Range	2–10	3–9	3–9	2–10		
Full Scale DQ (MSEL)	72.5(20.2)	91.8(15.0)	72.8(15.2)	59.4(13.8)	58.20	<0.001
Mean (SD), Range	27.7–128.0	57.3–128.0	50.7–104.6	27.6–94.5		
Fine Motor Skills (MSEL)	26.9(5.0)	31.5(5.2)	26.6(3.6)	23.9(2.8)	42.84	<0.001
Mean (SD), Range	19–45	22–45	21–36	19–30		
Receptive Language (MSEL)	23.2(7.8)	30.8(5.4)	24.2(4.6)	17.7(5.4)	69.97	<0.001
Mean (SD), Range	11–45	14–45	14–31	11–31		
ADOS-2 (CSS)	7.0(1.7)	6.8(1.6)	7.0(1.5)	7.1(1.9)	0.32	0.72
Mean (SD), Range	4–10	4–10	4–10	4–10		
Autistic Features (SRS)	70.3(10.8)	68.3(10.5)	70.2(10.6)	71.6(11.6)	1.17	0.31
Mean (SD), Range	47–97	51–85	51–88	47–97		
DWI Scan (*n* [% of sample])	86 (70%)	33 (85%)	15 (60%)	38 (66%)	5.30^a^	0.07
Scanner System (n Post-upgrade[%])	41 (48%)	18 (54%)	9 (60%)	14 (37%)	3.32^a^	0.19
Head motion (RMS)	0.11(0.07)	011(0.10)	0.09(0.02)	0.11(0.04)	1.44	0.24
Mean (SD), Range	0.05–0.65	0.06–0.65)	0.05–0.13	0.5–0.27		
**Follow-up**						
Age (Months)	70.1(10.2)	70.1(10.2)	69.3(13.1)	63.8(8.1)	5.09	0.007
Mean (SD), Range	54.0–101.7	50.11–101.7	55.2–112.4	51.1–101.7		
EOWPVT raw score	53.1(24.1)	73.6(15.7)	56.8(14.6)	37.8(21.3)	49.00	<0.001
Mean (SD), Range	0–109	47–109	24–78	0–87		
Inter-visit Interval (Months)	30.5(7.9)	31.2(9.1)	32.0(9.9)	29.3(5.7)	1.27	0.28
Mean (SD), Range	23.1–64.3	23.7–64.3	23.1–60.2	23.6–57.1		
EOWPVT Change Rate	1.9(0.6)	1.2(0.4)	1.2(0.5)	1.2(0.7)	0.14	0.86
Mean (SD), Range	−0.1–2.8	0.0–2.0	0.1–1.9	−0.1–2.8		
Full Scale IQ (DAS-II)	91.1(26.0)	109.3(11.6)	92.8(23.5)	78.0(26.6)	24.52	<0.001
Mean (SD), Range	20.7–133.0	87.0–133.0	41.0–130.0	20.7–116.0		
ADOS-2 (CSS)	6.8(2.3)	5.7(2.4)	7.4(1.4)	7.2(2.2)	6.90	0.002
Mean (SD), Range	1–10	1–10	5–10	5–10		
Autistic Features (SRS)	71.1(11.9)	70.9(12.8)	73.6(13.9)	69.9(10.2)	0.51	0.60
Mean (SD), Range	44–98	45–98	46–93	44–85		

*EOWPVT*, Expressive One Word Picture Vocabulary Test; *VABS-II*, Vineland Adaptive Behavior Scale; *MSEL*, Mullen Scales of Early Learning; *ADOS-2*, Autism Diagnostic Observation Schedule, Version 2; *SRS*, Social Responsiveness Scale; *DWI*, Diffusion Weighted Imaging; *RMS*, Root Mean Squared Displacement of Head Motion; *DAS-II*, Differential Ability Scale-II.

^a^X^2^ statistic instead of F statistic.

### Ethics approval and consent to participate

All aspects of the study protocol were approved by the University of California Davis Institutional Review Board. Informed consent was obtained from the parent or guardian at both timepoints.

### Spoken vocabulary assessment

#### Expressive one word picture vocabulary test (EOWPVT)

The EOWPVT is an observational measure of spoken vocabulary, consisting of 109 pictures that test object, categorical, and symbolic vocabulary. Participants were encouraged to verbally label the subject of each picture, with additional prompts for actions or groups of items. Raw scores were utilized in analyses because they provide a broader range than the EOWPVT norm-referenced scores and therefore show greater distribution as well as specificity of ability and change in this neurodiverse dataset [32, 33]. 69 participants received the EOWPVT Version 3 [34] at both timepoints and 53 received EOWPVT Version 4 [35] at both timepoints. No significant group differences in EOWPVT scores were identified between participants with Version 3 and Version 4, so groups were collapsed.

#### Spoken vocabulary starting groups

Children were divided into three vocabulary groups based on their EOWPVT scores at Time 1. Children were placed in the ‘smaller starting spoken vocabulary’ group if their raw EOWPVT scores were 12 or below (*n* = 58). Given the administration procedures for the EOWPVT (i.e., stopping after 6 consecutive incorrect responses), a score of 12 or below could include producing words such as ‘*boat’*, ‘*tree’*, ‘*cat’*, ‘*bus’*, ‘*book’*, and ‘*duck’*. These early-learned nouns are produced by more than 85% of American English learning children by 30 months of age as documented by Wordbank [36]. If a child was speaking *only* these kinds of early-learned words at three years old, their vocabulary level would be comparable to a child of two years old or younger without developmental delays. Children were placed in the ‘larger starting spoken vocabulary’ group if their raw EOWPVT scores were 26 or higher (*n* = 39). These children produced words that referred to categories of objects (e.g., producing ‘*animals*’ for a picture of a cow, bear, turkey, and giraffe). The ability to use category or superordinate labels is significantly more advanced than expected for a three-year-old without developmental delays [37–39]. Children with EOWPVT raw scores of 13 through 25 were placed into the ‘average starting spoken vocabulary’ group (*n* = 25); they could produce words like ‘*leaf*’, ‘*kite*’ and ‘*basket*’, all of which are more advanced than what would be expected for two-year-olds without developmental delays yet still designate individual objects at the basic/single object level. Hence, their word production is appropriate for their chronological age level.

#### Spoken vocabulary change

EOWPVT rate of change was calculated by dividing the difference in EOWPVT raw scores by the difference in the age (months) between Time 1 and Follow-up.

### Additional behavioral measures acquired at time 1

#### Fine motor (Mullen Scales of Early Learning [MSEL])

The MSEL is a standardized assessment of early development from 0 to 68 months [40] that includes a FM skills assessment. Time 1 MSEL FM skill raw scores were used to mitigate floor-effects and capture the full scope of variation that exists in this specific dataset. Higher MSEL FM scores indicate better performance. The MSEL was only administered at Time 1 and therefore longitudinal metrics cannot be derived.

#### Joint attention (ADOS-2)

JA was measured using an ADOS-2-derived JA factor for modules 1 and 2 which is comprised of five items taken from the Social Affect subscale including: pointing, gestures, initiating joint attention, showing, and unusual eye contact [41–43]. The JA factor is distinct from the Social Affect score and Calibrated Severity Score (CSS) and primarily reflects initiating JA behaviors. Following ADOS-2 scoring rules, all item scores receiving a 3 were converted to 2. Next, all items were summed to obtain a total *joint attention factor score* with a range of 0–10, with a score of 0 indicating strong joint attention skills.

### Image acquisition and processing

MRI scans were acquired at the University of California-Davis Imaging Research Center on a 3T Siemens Trio MRI system (Erlangen, Germany) using an eight-channel head coil. All scans were performed during natural nocturnal sleep without sedation [25] between 2007 and 2022. In 2009, the MRI scanner was upgraded to a TIM system (see Supplementary Methods).

Diffusion weighted MRI was acquired in 30 independent directions along with five interleaved non-diffusion weighted images (1.9mm^3^ resolution, TR = 8500 ms, TE = 91 ms, b = 700, 243x243x137mm FOV). Images were preprocessed using MRtrix3 pipelines [44] that utilized FSL v5.0.11. Preprocessing corrected for artifacts caused by noise [45], Gibbs ringing [46, 47], eddy currents, and within and between volume motion distortion [48, 49]. Preprocessed images were fitted for diffusion tensors and maps of fractional anisotropy (FA), mean diffusivity (MD), radial diffusivity (RD) and axial diffusivity (AD) were calculated [50, 51]. Preprocessed volumes were visually screened to ensure quality. Head motion was quantified using the root-mean-square (RMS) displacement calculated during preprocessing and used as a covariate in analyses. Scan quality metrics did not statistically differ among spoken vocabulary groups (Supplementary Table 1).

Bilateral white matter projections were delineated using TractSeg [52]. Native space bilateral segmentations of the inferior longitudinal fasciculus (ILF), arcuate fasciculus (AF), corticospinal tract (CST), and uncinate fasciculus (UF) were further parcellated into three equal-length segments using a tractometry approach [53] (Figure S1). Measurement of diffusion parameters along tracts can provide valuable insights as calculating the average diffusion metric across large tracts that traverse the brain can obscure individual differences. This approach has been used in prior developmental studies, demonstrating that language-related effects localize to specific tract subregions that align with the three segmentations evaluated in the present study [8, 24, 26]. Supplementary analyses using a finer-grained 9-segment scheme (Figure S2) revealed spatially circumscribed effects that converged with the three-segment findings reported in the main text. Thus, dividing each tract into three segments reflected a hypothesis-driven approach that balanced anatomical specificity with statistical stability and interpretability in this pediatric sample. Median DTI metrics from each of the three segments within each tract of interest were used in analyses.

### Statistical analyses

All analyses were conducted in R (v4.0.3), controlling for age, sex, and EOWPVT raw score at Time 1. Primary analyses characterized predictors of spoken vocabulary development within the group of autistic children with small starting vocabularies. Secondary analyses tested the specificity and generalizability of effects across the broader sample. Follow-up analyses tested models across the full sample, regardless of starting vocabulary level.

#### Behavioral predictors

General linear models (GLMs) tested whether Time 1 JA and FM skills independently or jointly predicted EOWPVT change, first in the smaller starting vocabulary group and then across the full sample using group-by-behavior interaction terms. Model fit was assessed via AIC and ANOVA.

#### Neurobiological predictors

To assess whether Time 1 DTI metrics (residualized for head motion) of language-associated tracts predicted EOWPVT change rates, separate GLMs were run for each DTI parameter (FA, MD, AD, RD) across 3 segments in 8 tracts. False discovery rate (FDR) correction was applied within each DTI parameter. Analyses were first performed within the smaller starting vocabulary group and then across the whole sample including a group-by-behavior interaction term. DTI measures in tract segments that were significantly associated with EOWPVT change in the smaller starting vocabulary group were used in subsequent analyses (i.e., segments with significant DTI parameter main effects in the smaller starting vocabulary group and significant DTI parameter-by-language group interaction effects that were driven by the smaller starting vocabulary group).

#### Combined models

We evaluated whether combining behavioral (JA, FM) and neural (left occipital ILF FA, left central AF FA, left inferior CST FA) predictors improved model fit in the smaller vocabulary group. Joint models were compared to behavioral- and brain-only models using AIC and ANOVA. Post-hoc path analyses explored mediation effects.

## Results

### Spoken vocabulary heterogeneity in autistic children

**Fig. 1:**
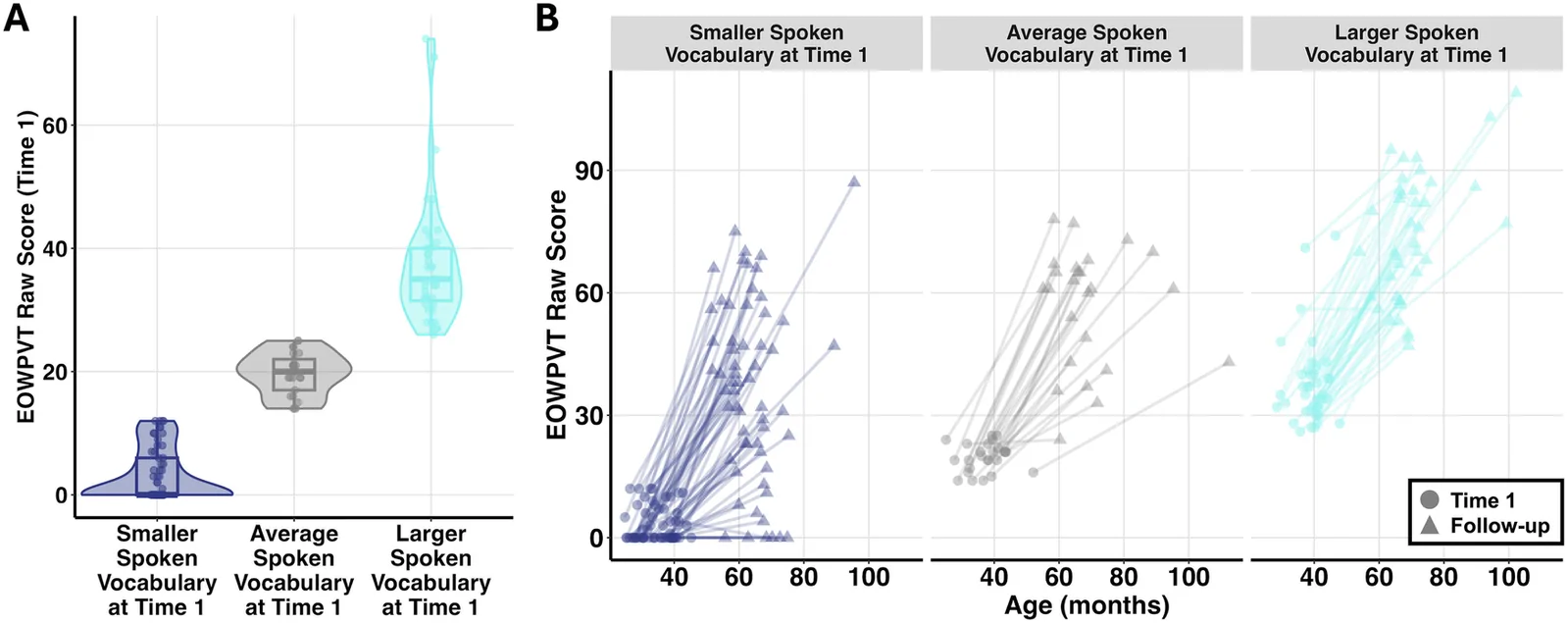
Starting spoken vocabulary levels and rates of change. Autistic children were assigned to one of three spoken vocabulary groups based on their EOWPVT raw scores at Time 1. Panel **A** shows the distribution of EOWPVT raw scores at Time 1 within each group. Panel **B** shows the EOWPVT raw score change for each child between Time 1 and Follow-up visits (which occurred approximately two years after Time 1).

47% of the autistic cohort (58 children) were in the smaller starting spoken vocabulary group (Table 1; Fig. 1A). At Time 1, this group had lower receptive language levels (F(2,115) = 69.9, *p* < 0.001) and was slightly younger (F(2,115) = 8.09, *p* < 0.001) than the other two groups. Proportionally, there were more males in the smaller starting vocabulary group compared to the other groups. Groups did not differ in autism features characterized by ADOS-CSS (F(2,115) = 0.32, *p* = 0.72) or SRS total t-scores (F(2,93) = 1.17, *p* = 0.31). At Follow-up, the larger starting vocabulary group had lower ADOS-CSS (less prominent autistic features) (F(2,115) = 6.90, *p* = 0.002; Table 1) than other groups.

Substantial variability in the rate of EOWPVT change was observed in all groups (Fig. 1B), with no significant group differences in the rate of change after accounting for Time 1 age, Time 1 EOWPVT raw scores, and sex (Levene’s Test F(2,115) = 2.71, *p* = 0.07).

### FM and JA skills predict rates of spoken vocabulary development in autistic children with smaller spoken vocabularies at Time 1

**Fig. 2:**
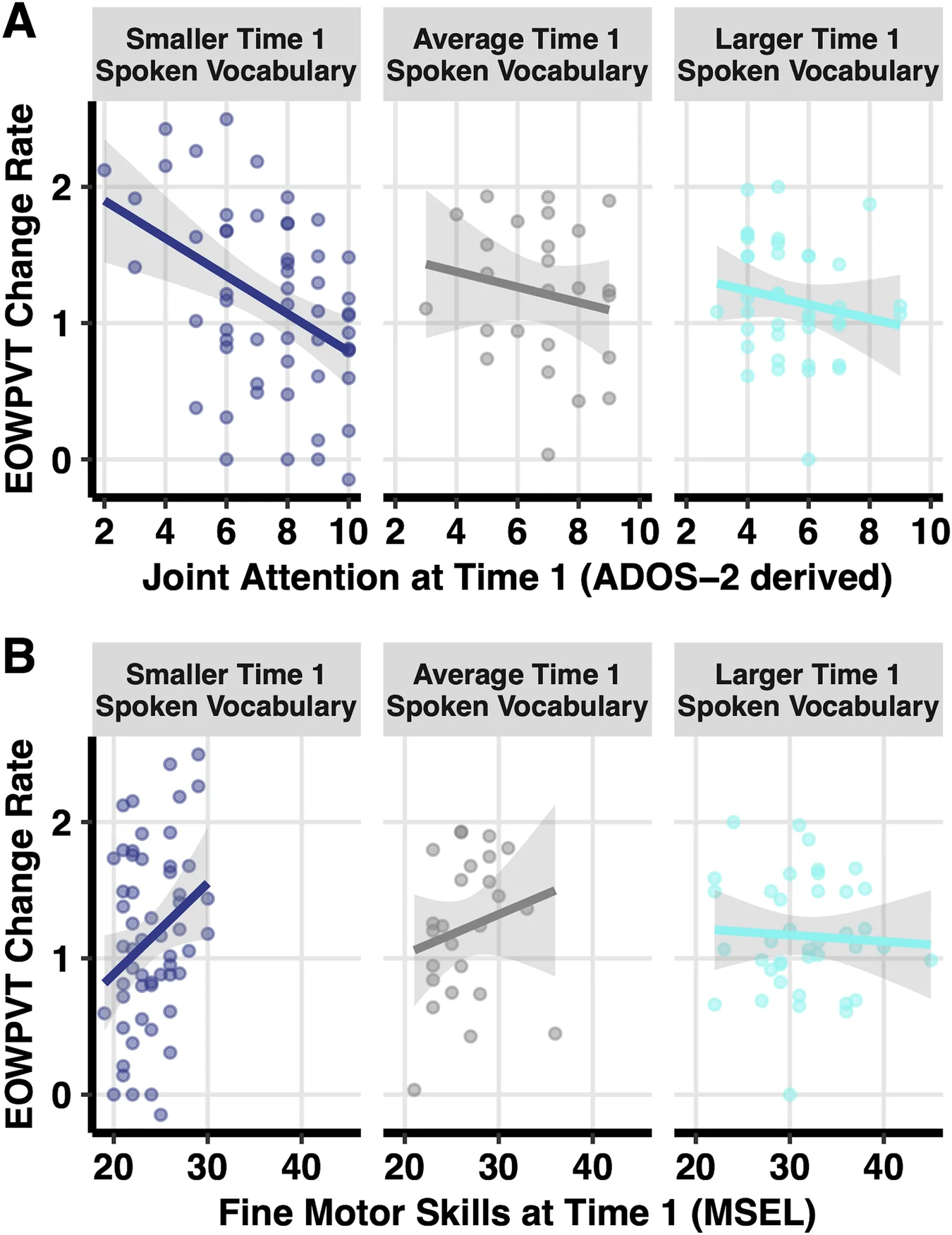
FM and JA skills predict EOWPVT rate of change in autistic children with smaller spoken vocabularies at Time 1. Scatter plots depict correlations between EOWPVT rate of change and Time 1 behavioral factors ([**A**] fine motor [FM] skills and [**B**] joint attention [JA] skills) in autistic children with smaller (dark blue), average (gray), and larger (light blue) spoken vocabularies at Time 1. Higher scores of the Mullen Scales of Early Learning (MSEL) FM measure indicate better performance. Lower scores on the ADOS-2-derived JA measure indicate better performance.

Within the smaller starting vocabulary group, better Time 1 FM skills predicted faster EOWPVT change rates (β = 0.09, t(52) = 2.72, *p* = 0.01) (Table S2). While this association was consistent across the full sample (Figure S3), the effect was strongest in the smaller vocabulary group, as indexed by a significant group-by-Time 1 FM skill interaction effect when predicting rates of EOWPVT change across the full sample (Fig. 2A; Table S3).

Better Time 1 JA was also predictive of faster EOWPVT change rate in the smaller starting vocabulary group (β = −0.11, t(52) = −2.82, *p* = 0.007) (Table S4). This association was consistent across the full sample (Figure S4) and, unlike the FM analysis, it had similar predictive power across groups as indexed by a non-significant group-by-Time 1 JA interaction effect when predicting EOWPVT change rate (Fig. 2B; Table S5, Figure S3).

To determine whether together FM and JA could predict EOWPVT change rate in the smaller starting vocabulary group better than only a single behavioral parameter, secondary analyses tested a model including both Time 1 FM and JA. Results of this model revealed significant main effects of both Time 1 FM skills and Time 1 JA skills, indicating that each parameter contributed unique power to predict spoken vocabulary change (Table S6). Model comparison demonstrated that this combined model of behavioral variables accounted for significantly more variance than models that included a single variable, either FM (ANOVA comparison: F(1,52) = 6.37, *p* = 0.01) or JA (ANOVA comparison: F(1,52) = 6.47, *p* = 0.01) (Table 2), suggesting a stronger model fit despite a statistical penalty for the additional parameters.

**Table 2:** Comparison of models predicting EOWPVT change rate autistic children with smaller spoken vocabularies at Time 1.

Model	Model F	Multiple R-squared	Adj. R-Squared	AIC	BIC
**Behavior Only Models**					
Fine Motor	5.95	0.26	0.21	104.6	114.8
Joint Attention	5.91	0.25	0.21	104.7	114.9
Fine motor + Joint Attention	6.51	0.34	0.29	100.0	112.2
**Brain Only Models**					
ILF FA, Left Occipital Segment	3.82	0.25	0.18	75.4	83.7
AF FA, Left Central Segment	2.01	0.15	0.07	80.2	88.5
CST FA, Left Inferior Segment	1.23	0.10	0.02	82.5	90.8
**Behavior + Brain Models**					
FM + JA + ILF FA, Left Occipital Segment	11.78	0.64	0.59	50.5	62.1
FM + JA + AF FA, Left Central Segment	7.70	0.54	0.47	60.3	71.9
FM + JA + CST FA, Left Inferior Segment	7.76	0.54	0.47	60.1	71.8

Lower BIC and lower AIC represent stronger model fit.

*EOWPVT*, Expressive One Word Picture Vocabulary Test; *FM*, Fine Motor; *JA*, Joint Attention; *ILF*, Inferior Longitudinal Fasciculus; *AF*, Arcuate Fasciculus; *CST*, Corticospinal Tract; *FA*, Fractional Anisotropy.

### White matter microstructure predicts rates of spoken vocabulary development in autistic children with smaller spoken vocabularies at Time 1

**Fig. 3:**
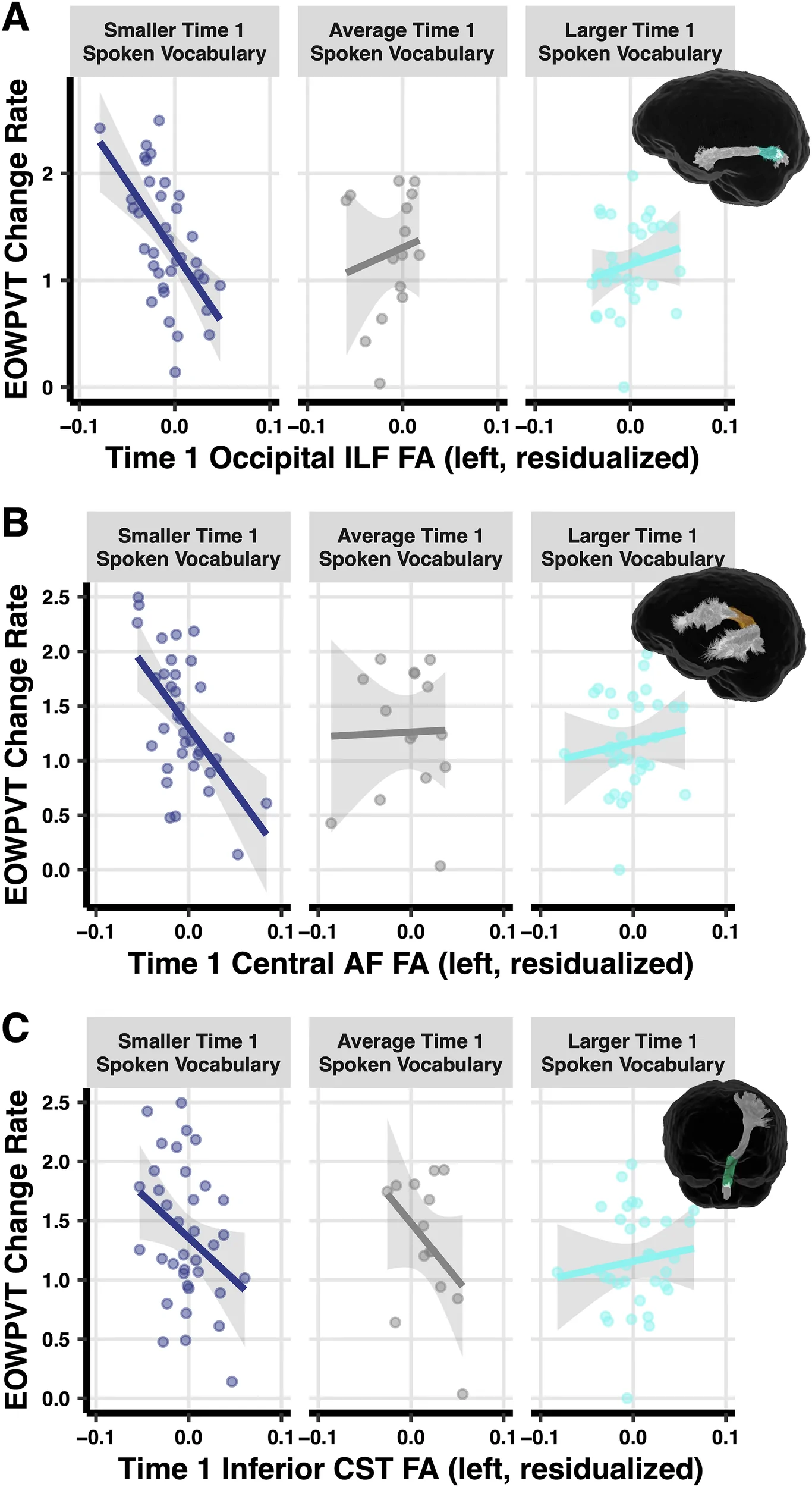
White matter microstructural properties predict EOWPVT rate of change in autistic children with smaller spoken vocabularies at Time 1. Scatter plots depict correlations between EOWPVT rate of change and Time 1 fractional anisotropy (FA) of the (**A**) occipital inferior longitudinal fasciculus (ILF), (**B**) central arcuate fasciculus (AF), and (**C**) inferior corticospinal tract (CST) in autistic children with smaller (dark blue), average (gray), and larger (light blue) spoken vocabularies at Time 1.

Within the smaller starting vocabulary group, EOWPVT change rate was negatively associated with Time 1 FA in the bilateral occipital ILF, left central AF, and bilateral inferior CST (Table S7). After FDR correction, relationships within the left posterior ILF remained significant (β = −12.84, t(32) = 3.50, *p* = 0.001, FDR *p* = 0.03). While these effects were largely consistent across the sample (Figure S4), effects were strongest in the smaller starting vocabulary group in several regions. Specifically, significant group-by-FA interaction effects were observed in the left posterior ILF, bilateral AF (all segments), and left inferior CST after correction for multiple comparisons (Table S8). In the left posterior ILF, left central AF, and left inferior CST, interaction effects were driven by negative correlations in the smaller starting vocabulary group and non-significant correlations in the average and larger starting vocabulary groups (Fig. 3; Table S9). MD, RD, and AD metrics corroborated FA findings at trend-levels but did not pass FDR correction (Tables S10-S12).

### White matter microstructure and behavior together best predict rates of spoken vocabulary development in autistic children with smaller spoken vocabularies at Time 1

**Fig. 4:**
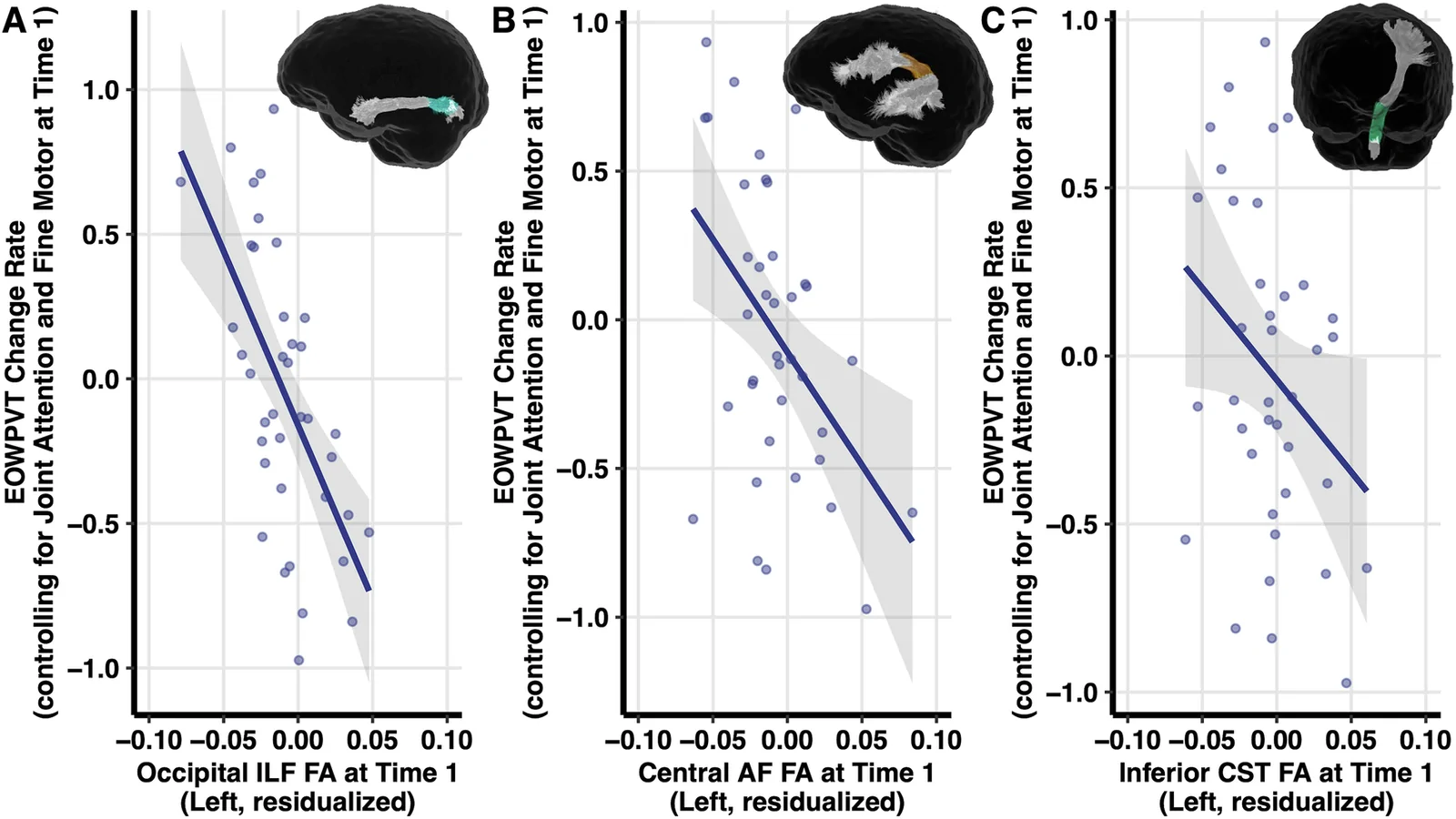
Time 1 white matter microstructure strongly predicts EOWPVT rates of change in autistic children with smaller spoken vocabularies at Time 1 after controlling for Time 1 FM and JA skills. Scatter plots depict correlations between EOWPVT rate of change and Time 1 fractional anisotropy (FA) of the (**A**) occipital inferior longitudinal fasciculus (ILF), (**B**) central arcuate fasciculus (AF), and (**C**) inferior corticospinal tract (CST) in autistic children with smaller spoken vocabularies at Time 1 after accounting for effects of joint attention [JA] and fine motor [FM] skills.

Post-hoc analyses within the smaller starting vocabulary group determined whether Time 1 brain metrics contributed explanatory information above and beyond behavioral variables (Time 1 JA and FM skills). Combined brain and behavior models were constructed for each white matter segment that showed significant associations with EOWPVT change rates in the smaller spoken vocabulary group (FA in left posterior ILF, central AF, and inferior CST). In all segments, the combined model predicted EOWPVT change rates better than both the brain-alone and behavior-alone models, accounting for roughly half of the model variance after adjusting for model size (Table 2). In all cases, FA, FM, and JA were each significant predictors of EOWPVT change rates, indicating that each of these three variables accounted for unique model variance (Fig. 4; Table S13). Follow-up path analyses found no significant mediating effects of Time 1 microstructure on associations between Time 1 behavior (FM and JA) and EOWPVT change rates (Table S14).

## Discussion

The present study examined brain and behavioral predictors of spoken vocabulary development over a two-year period in young autistic children with a wide range of spoken language abilities. Nearly half of the sample (47%) had limited-to-no spoken vocabularies at approximately age three years. Many of these children demonstrated substantial gains in spoken vocabulary across timepoints, a pattern that was similarly observed among autistic children who had average or larger vocabularies at age three. Further, FM skills, JA skills, and white matter microstructure in language processing and speech-related tracts at age three were all associated with spoken vocabulary development, with the most robust effects observed in autistic children with smaller initial vocabularies. Specifically, for autistic children with smaller spoken vocabularies at three, the amount of spoken vocabulary gain over time was independently associated with better Time 1 FM skills and Time 1 JA skills, as well as with lower Time 1 FA in tracts associated with language processing (ILF, AF) and speech production (CST). Importantly, statistical models that simultaneously leveraged both behavioral (FM and JA) and brain imaging (FA) metrics best accounted for the heterogeneity in spoken vocabulary change rates. Together, findings inform our understanding of early factors that contribute to spoken language development and suggest that specific neurobiological characteristics provide salient developmental information above and beyond developmental behavioral measures alone.

Notably, spoken vocabulary development was highly variable over the two-year span both across the full autistic sample of children and within the group of children with smaller initial vocabularies, ranging from no change in EOWPVT scores to average increases of 2.8 EOWPVT points/month. The high change rates in this subset of autistic children corroborate previous work and are reflective of substantial gains in spoken vocabulary, representing meaningful improvements in language ability during a critical developmental period [37, 38, 54]. Importantly, spoken vocabulary production reflects only one modality through which language is expressed. Spoken vocabulary scores may be influenced by multiple underlying processes, including receptive language ability, lexical-semantic knowledge, and speech motor control. Therefore, changes in spoken vocabulary skills may reflect changes in comprehension, speech production mechanisms, or the integration of these systems.

We also found that the extent to which spoken vocabulary improved between visits was associated with both early childhood FM and JA skills. Findings corroborate previous work and theoretical models of a spoken language developmental cascade [2, 16, 19]. Specifically, findings suggest that together, early proficiency in the coordinated manipulation of objects (FM skills) and the ability to engage with and direct the attention of others (JA) facilitate important interactions with the human and physical environment and contribute to spoken language development. These findings also underscore the importance of considering both motor and social-communicative abilities, especially attention sharing, in early assessments and interventions aimed at supporting language development in autistic children.

Additionally, we found that early childhood white matter microstructure of the left occipital ILF, left central AF, and left inferior CST (but not UF) predicted rates of spoken vocabulary change specifically in the group with smaller spoken vocabularies at age three. Previous studies have reported associations among concurrent measures of language ability and microstructure in autistic populations from similar segments of the ILF [8, 24], AF [26, 55], and CST [8]. These loci may be particularly salient because they each represent critical junctions for language information transfer. The occipital ILF is associated with integrating visual and auditory information essential for word learning [56], the central AF is associated with Geschwind’s territory in the temporoparietal junction linked to language comprehension [57], and the inferior CST directly projects to brainstem nuclei and cranial nerves that innervate muscles necessary for speech production [27, 56]. Notably, the central AF segment examined includes portions of both the long direct segment and the anterior indirect segment. The long direct segment has been implicated in articulation and phonological processing, whereas the anterior indirect segment is more strongly associated with semantic knowledge and language comprehension. Because our tract segmentation did not fully dissociate these components, we cannot determine whether associations with spoken vocabulary primarily reflect speech motor, semantic, or integrative language processes. Collectively, results suggest that early childhood differences in these regions provide information regarding the potential for future speech development of spoken language.

Further*, lower* FA values in specific white matter regions were associated with *faster* rates of spoken vocabulary change in autistic children with smaller spoken vocabularies at age three. This negative association between early FA and later spoken language ability has also been demonstrated in other pediatric clinical and non-clinical populations [58–60] and corroborates previous work examining FA and behavioral development in autistic populations [61–63]. Previously, we showed that on a group level, at age three, autistic children have higher FA than non-autistic peers in several white matter pathways but experience slower rates of white matter development, leading to lower FA by age five [61]. We also found that on an individual level, autistic children whose autism features became less prominent (ADOS CSS score reductions) between ages three and five had lower FA at age three than those whose autism features became more prominent (ADOS CSS score increases) [61]. The results of the present work, which employed a partially overlapping sample, support previous findings by suggesting that autistic children with lower FA at age three may also experience the greater gains in spoken vocabulary by age five. In these children, both language and white matter may develop at a more rapid pace, resulting in the largest speech and global developmental changes. This could indicate that spoken language and overall developmental trajectories in young autistic children are closely related to the time course of early white matter maturation and thus provide a potential brain-based mechanism supporting the rapid spoken vocabulary development observed in a subset of young autistic children with early speech delays [4].

To our knowledge, this is the first study to demonstrate the utility of leveraging combined brain imaging and behavioral metrics to predict longitudinal spoken vocabulary development in young autistic children. We found that for young autistic children with the smallest starting vocabularies, spoken vocabulary development over the subsequent two years was best predicted by a combination of behavioral and brain imaging factors and that each of these measures contributed independently to spoken vocabulary development. Although the combined model was less parsimonious than single-predictor models, model fit indices indicated significantly improved explanatory value, supporting the inclusion of both behavioral and neurobiological predictors when characterizing developmental heterogeneity. This suggests that aspects of early brain structure provide additional explanatory power that cannot be accounted for by exclusively behavioral predictors of language outcomes. While most individual predictors accounted for modest but meaningful proportions of variance in spoken vocabulary change, FA of the left occipital ILF accounted for approximately 20% of the variance in vocabulary change, suggesting that this tract may play a particularly salient role in early spoken vocabulary development. Collectively, these findings provide further information to the existing behaviorally-based developmental cascade of spoken language in early childhood; specifically, that spoken language development is dependent on foundational motoric and social-attentional developmental skills *and* white matter maturation. Future work may focus on more specific evaluations of these developmental time courses in autistic children with language delays and may be particularly relevant for individuals who support children’s communication development.

This study should be interpreted in the context of both its limitations and strengths. While the EOWPVT is a measure of spoken vocabulary, there are certainly additional aspects of spoken language and language comprehension that are foundational for communication. Although spoken vocabulary provides important clinical information, future research would benefit from incorporating more aspects of syntactic, pragmatic, and deeper lexical knowledge as well as evaluation of nuanced language comprehension phenotypes (e.g., [64]) to examine how trajectories of expressive and receptive language development relate to the maturation of white matter pathways.

An additional potential limitation relates to the trichotomization of children based on initial spoken vocabulary. While categorizing continuous variables can reduce statistical power and inflate Type I error under certain conditions, our grouping strategy was hypothesis-driven, grounded in developmental theory and prior empirical work distinguishing emergent or minimally speaking children from peers with higher early language levels [8]. Moreover, subgroup analyses were complemented by continuous-variable models, which yielded converging results and supported the robustness of the primary conclusions. Nevertheless, replication in larger samples and the application of alternative analytic approaches, such as nonlinear growth models or mixture modeling, will be critical for corroborating subgroup-specific effects and refining our understanding of heterogeneity in spoken language development.

Furthermore, our diffusion imaging sequence introduces restrictions in terms of diffusion modeling and interpretation. This single-shell acquisition, which has stayed consistent for longitudinal harmony since the start of data collection in 2006, only allows for the evaluation of white matter microstructure within the context of traditional diffusion tensor-based metrics. While this may limit the interpretation of which neurobiological mechanisms are at play during this critical time of white matter development, the use of tractometry-based approaches for analysis allows us to localize our findings to specific segments of large white matter pathways that traverse the brain. Lastly, our ability to acquire brain scans and behavioral data from young autistic children with little-to-limited speech and greater developmental delays gives us a unique ability to evaluate brain-behavior associations in a population commonly excluded from neuroimaging research.

In sum, this work provides new insight into the highly variable trajectories of spoken vocabulary development in young autistic children. Although children differed substantially in their starting language abilities, all groups demonstrated change in spoken vocabulary over time, including those with the smallest initial vocabularies. Across the two-year period, the rate of this change was best explained by a combination of early behavioral abilities and white matter microstructure, particularly among children with early speech delays. These findings suggest that early spoken language development reflects the interaction of motor, social-attentional, and neurobiological processes. By demonstrating that brain-based metrics contribute unique explanatory value beyond behavioral measures alone, this study extends existing developmental cascade models of language by incorporating early neural characteristics into our understanding of language acquisition in autism. Together, these results highlight the importance of considering both behavioral and neurobiological variability when characterizing developmental trajectories and provide a foundation for future research examining how early developmental supports and interventions may interact with emerging brain systems to promote communication outcomes.

## Supplementary information


Supplemental Material


## Data Availability

Analysis code and original data can be made available upon reasonable request.

## References

[1] Thurm A, Manwaring SS, Swineford L, Farmer C. Longitudinal study of symptom severity and language in minimally verbal children with autism. Child Psychology Psychiatry. 2015;56:97–104. 10.1111/jcpp.12285. PMC4581593 24961159

[2] Bal VH, Fok M, Lord C, Smith IM, Mirenda P, Szatmari P, et al. Predictors of longer-term development of expressive language in two independent longitudinal cohorts of language-delayed preschoolers with Autism Spectrum Disorder. Child Psychology Psychiatry. 2020;61:826–35. 10.1111/jcpp.13117. PMC7028445 31429087

[3] Gernsbacher MA, Morson EM, Grace EJ. Language Development in Autism. Neurobiology of Language [Internet]. Elsevier; 2016 [cited 2024 Oct 25]. p. 879–86. 10.1016/B978-0-12-407794-2.00070-5

[4] Smith V, Mirenda P, Zaidman-Zait A. Predictors of expressive vocabulary growth in children with autism. J Speech Lang Hear Res. 2007;50:149–60. 10.1044/1092-4388(2007/013). 17344556

[5] Tek S, Mesite L, Fein D, Naigles L. Longitudinal analyses of expressive language development reveal two distinct language profiles among young children with autism spectrum disorders. J Autism Dev Disord. 2014;44:75–89. 10.1007/s10803-013-1853-4. 23719855 PMC4557801

[6] Pickles A, Anderson DK, Lord C. Heterogeneity and plasticity in the development of language: a 17-year follow-up of children referred early for possible autism. Child Psychology Psychiatry. 2014;55:1354–62. 10.1111/jcpp.12269. 24889883

[7] Abdelaziz A, Wagner M, Naigles LR. Associations between joint attention, supported joint engagement and language in TD children and children with ASD: potential sources of individual and group differences in language outcomes. Language Learning and Development. 2024;1–31. 10.1080/15475441.2024.2336047 PMC11737634 39830169

[8] Naigles LR, Johnson R, Mastergeorge A, Ozonoff S, Rogers SJ, Amaral DG, et al. Neural correlates of language variability in preschool-aged boys with autism spectrum disorder: Neural correlates of language in preschoolers. Autism Research. 2017;10:1107–19. 10.1002/aur.1756. 28301102 PMC5548458

[9] LeBarton ES, Landa RJ. Infant motor skill predicts later expressive language and autism spectrum disorder diagnosis. Infant Behavior and Development. 2019;54:37–47. 10.1016/j.infbeh.2018.11.003. 30557704

[10] Tager-Flusberg H, Kasari C. Minimally verbal school-aged children with autism spectrum disorder: the neglected end of the spectrum. Autism Research. 2013;6:468–78. 10.1002/aur.1329. 24124067 PMC3869868

[11] Leonard HC, Bedford R, Pickles A, Hill EL. Predicting the rate of language development from early motor skills in at-risk infants who develop autism spectrum disorder. Research in Autism Spectrum Disorders. 2015;13–14:15–24. 10.1016/j.rasd.2014.12.012. 31410102 PMC6692163

[12] Naigles LR, Fein D Looking through their eyes: Tracking early language comprehension in ASD. In: Naigles LR, editor. Innovative investigations of language in autism spectrum disorder [Internet]. Berlin: Walter de Gruyter GmbH; 2017 [cited 2025 Feb 24]. p. 49–69. 10.1037/15964-004

[13] Thomas RP, Wittke K, Blume J, Mastergeorge AM, Naigles L. Predicting language in children with ASD using spontaneous language samples and standardized measures. J Autism Dev Disord. 2023;53:3916–31. 10.1007/s10803-022-05691-z. 35930209

[14] Blume J, Wittke K, Naigles L, Mastergeorge AM. Language growth in young children with autism: interactions between language production and social communication. J Autism Dev Disord. 2021;51:644–65. 10.1007/s10803-020-04576-3. 32588273

[15] Bruyneel E, Demurie E, Warreyn P, Roeyers H. The mediating role of joint attention in the relationship between motor skills and receptive and expressive language in siblings at risk for autism spectrum disorder. Infant Behavior and Development. 2019;57:101377. 10.1016/j.infbeh.2019.101377. 31541867

[16] Iao L-S, Shen C-W, Wu C-C. A longitudinal study of joint attention, motor imitation and language development in young children with autism spectrum disorder in Taiwan. J Autism Dev Disord. 2024;54:2651–62. 10.1007/s10803-023-05950-7. 37142905

[17] Salo VC, Rowe ML, Reeb-Sutherland B. Exploring infant gesture and joint attention as related constructs and as predictors of later language. Infancy. 2018;23:432–52. 10.1111/infa.12229. 29725273 PMC5927593

[18] Butler LK, Tager-Flusberg H. Fine motor skill and expressive language in minimally verbal and verbal school-aged autistic children. Autism Research. 2023;16:630–41. 10.1002/aur.2883. 36578205 PMC10320849

[19] Iverson JM. Developing language in a developing body: the relationship between motor development and language development. J Child Lang. 2010;37:229–61. 10.1017/S0305000909990432. 20096145 PMC2833284

[20] Fedorenko E, Thompson-Schill SL. Reworking the language network. Trends in Cognitive Sciences. 2014;18:120–6. 10.1016/j.tics.2013.12.006. 24440115 PMC4091770

[21] Friederici AD, Gierhan SM. The language network. Current Opinion in Neurobiology. 2013;23:250–4. 10.1016/j.conb.2012.10.002. 23146876

[22] Simonyan K, Fuertinger S. Speech networks at rest and in action: interactions between functional brain networks controlling speech production. Journal of Neurophysiology. 2015;113:2967–78. 10.1152/jn.00964.2014. 25673742 PMC4416556

[23] Li M, Wang Y, Tachibana M, Rahman S, Kagitani-Shimono K. Atypical structural connectivity of language networks in autism spectrum disorder: A meta-analysis of diffusion tensor imaging studies. Autism Research. 2022;15:1585–602. 10.1002/aur.2789. 35962721 PMC9546367

[24] Li M, Izumoto M, Wang Y, Kato Y, Iwatani Y, Hirata I, et al. Altered white matter connectivity of ventral language networks in autism spectrum disorder: An automated fiber quantification analysis with multi-site datasets. NeuroImage. 2024;297:120731. 10.1016/j.neuroimage.2024.120731. 39002786

[25] Nordahl CW, Simon TJ, Zierhut C, Solomon M, Rogers SJ, Amaral DG. Brief report: methods for acquiring structural mri data in very young children with autism without the use of sedation. J Autism Dev Disord. 2008;38:1581–90. 10.1007/s10803-007-0514-x. 18157624 PMC4864596

[26] Olivé G, Slušná D, Vaquero L, Muchart-López J, Rodríguez-Fornells A, Hinzen W. Structural connectivity in ventral language pathways characterizes non-verbal autism. Brain Struct Funct. 2022;227:1817–29. 10.1007/s00429-022-02474-1. 35286477 PMC9098538

[27] Morgan AT, Su M, Reilly S, Conti-Ramsden G, Connelly A, Liégeois FJ. A brain marker for developmental speech disorders. The Journal of Pediatrics. 2018;198:234–239.e1. 10.1016/j.jpeds.2018.02.043. 29705112

[28] Nordahl CW, Andrews DS, Dwyer P, Waizbard-Bartov E, Restrepo B, Lee JK, et al. The autism phenome project: toward identifying clinically meaningful subgroups of autism. Front Neurosci. 2022;15:786220. 10.3389/fnins.2021.786220. 35110990 PMC8801875

[29] Lord C, Risi S, Lambrecht L, Cook EH Jr., Leventhal BL, DiLavore PC, et al. The autism diagnostic observation schedule—generic: a standard measure of social and communication deficits associated with the spectrum of autism. Journal of Autism and Developmental Disorders. 2000;30:205–23. 10.1023/A:1005592401947. 11055457

[30] Lord C, Rutter M, Risi S, Gotham K, Bishop S Autism diagnostic observation schedule–2nd edition (ADOS-2) Manual (Part I): Modules 1–4. Los Angeles, CA: Western Psychological Corporation; 2012.

[31] Rutter M, Le Couteur A, Lord C Autism Diagnostic Interview Revised. Los Angeles, CA: Western Psychological Services; 2003.

[32] Landa RJ, Gross AL, Stuart EA, Faherty A. Developmental trajectories in children with and without autism spectrum disorders: the first 3 years. Child Development. 2013;84:429–42. 10.1111/j.1467-8624.2012.01870.x. 23110514 PMC4105265

[33] Perryman TY, Carter AS, Messinger DS, Stone WL, Ivanescu AE, Yoder PJ. Brief report: parental child-directed speech as a predictor of receptive language in children with autism symptomatology. J Autism Dev Disord. 2013;43:1983–7. 10.1007/s10803-012-1725-3. 23203905 PMC3651804

[34] Brownell R Expressive One-Word Picture Vocabulary Test. 3rd ed. Academic Therapy Publications; 2000.

[35] Martin NA, Brownell R Expressive One-Word Picture Vocabulary Test. 4th ed. Academic Therapy Publications; 2011.

[36] Frank MC, Braginsky M, Yurovsky D, Marchman VA. Wordbank: an open repository for developmental vocabulary data. J Child Lang. 2017;44:677–94. 10.1017/S0305000916000209. 27189114

[37] Greenfield DB, Scott MS. Individual differences in preschool children’s knowledge of superordinate categories. Journal of Applied Developmental Psychology. 1985;6:321–34. 10.1016/0193-3973(85)90007-3.

[38] Gülgöz S, Gelman SA. Children’s recall of generic and specific labels regarding animals and people. Cognitive Development. 2015;33:84–98. 10.1016/j.cogdev.2014.05.002. 25598575 PMC4292889

[39] Mervis CB, Crisafi MA. Order of acquisition of subordinate-, basic-, and superordinate-level categories. Child Development. 1982;53:258 10.2307/1129660.

[40] Mullen EM Mullen Scales of Early Learning. Circle Pines, MN: American Guidance Service, Inc; 1995.

[41] Gotham K, Risi S, Pickles A, Lord C. The autism diagnostic observation schedule: revised algorithms for improved diagnostic validity. J Autism Dev Disord. 2007;37:613–27. 10.1007/s10803-006-0280-1. 17180459

[42] Oosterling I, Roos S, De Bildt A, Rommelse N, De Jonge M, Visser J, et al. Improved diagnostic validity of the ADOS revised algorithms: a replication study in an independent sample. J Autism Dev Disord. 2010;40:689–703. 10.1007/s10803-009-0915-0. 20148299 PMC2864898

[43] Gotham K, Risi S, Dawson G, Tager-Flusberg H, Joseph R, Carter A, et al. A Replication of the Autism Diagnostic Observation Schedule (ADOS) revised algorithms. Journal of the American Academy of Child & Adolescent Psychiatry. 2008;47:642–51. 10.1097/CHI.0b013e31816bffb7. 18434924 PMC3057666

[44] Tournier J-D, Smith R, Raffelt D, Tabbara R, Dhollander T, Pietsch M, et al. MRtrix3: A fast, flexible and open software framework for medical image processing and visualisation. NeuroImage. 2019;202:116137. 10.1016/j.neuroimage.2019.116137. 31473352

[45] Veraart J, Fieremans E, Novikov DS. Diffusion MRI noise mapping using random matrix theory: Diffusion MRI Noise mapping. Magn Reson Med. 2016;76:1582–93. 10.1002/mrm.26059. 26599599 PMC4879661

[46] Kellner E, Dhital B, Kiselev VG, Reisert M. Gibbs-ringing artifact removal based on local subvoxel-shifts: Gibbs-Ringing Artifact Removal. Magn Reson Med. 2016;76:1574–81. 10.1002/mrm.26054. 26745823

[47] Veraart J, Fieremans E, Jelescu IO, Knoll F, Novikov DS. Gibbs ringing in diffusion MRI: Gibbs Ringing in Diffusion MRI. Magn Reson Med. 2016;76:301–14. 10.1002/mrm.25866. 26257388 PMC4915073

[48] Andersson JLR, Graham MS, Drobnjak I, Zhang H, Filippini N, Bastiani M. Towards a comprehensive framework for movement and distortion correction of diffusion MR images: Within volume movement. NeuroImage. 2017;152:450–66. 10.1016/j.neuroimage.2017.02.085. 28284799 PMC5445723

[49] Andersson JLR, Sotiropoulos SN. An integrated approach to correction for off-resonance effects and subject movement in diffusion MR imaging. NeuroImage. 2016;125:1063–78. 10.1016/j.neuroimage.2015.10.019. 26481672 PMC4692656

[50] Jenkinson M, Beckmann CF, Behrens TEJ, Woolrich MW, Smith SMFSL. NeuroImage. 2012;62:782–90. 10.1016/j.neuroimage.2011.09.015. 21979382

[51] Smith SM, Jenkinson M, Woolrich MW, Beckmann CF, Behrens TEJ, Johansen-Berg H, et al. Advances in functional and structural MR image analysis and implementation as FSL. NeuroImage. 2004;23:S208–19. 10.1016/j.neuroimage.2004.07.051. 15501092

[52] Wasserthal J, Neher P, Maier-Hein KH. TractSeg - Fast and accurate white matter tract segmentation. NeuroImage. 2018;183:239–53. 10.1016/j.neuroimage.2018.07.070. 30086412

[53] Chandio BQ, Risacher SL, Pestilli F, Bullock D, Yeh F-C, Koudoro S, et al. Bundle analytics, a computational framework for investigating the shapes and profiles of brain pathways across populations. Sci Rep. 2020;10:17149. 10.1038/s41598-020-74054-4. 33051471 PMC7555507

[54] Rogers SJ, Yoder P, Estes A, Warren Z, McEachin J, Munson J, et al. A Multisite randomized controlled trial comparing the effects of intervention intensity and intervention style on outcomes for young children with autism. Journal of the American Academy of Child & Adolescent Psychiatry. 2021;60:710–22. 10.1016/j.jaac.2020.06.013. 32853704 PMC8057785

[55] Duan K, Eyler L, Pierce K, Lombardo MV, Datko M, Hagler DJ, et al. Differences in regional brain structure in toddlers with autism are related to future language outcomes. Nat Commun. 2024;15:5075. 10.1038/s41467-024-48952-4. 38871689 PMC11176156

[56] Dick F, Taylor Tierney A, Lutti A, Josephs O, Sereno MI, Weiskopf N. In vivo functional and myeloarchitectonic mapping of human primary auditory areas. Journal of Neuroscience. 2012;32:16095–105. 10.1523/JNEUROSCI.1712-12.2012. 23152594 PMC3531973

[57] Catani M, Mesulam M. The arcuate fasciculus and the disconnection theme in language and aphasia: History and current state. Cortex. 2008;44:953–61. 10.1016/j.cortex.2008.04.002. 18614162 PMC2740371

[58] Dubner SE, Rose J, Bruckert L, Feldman HM, Travis KE. Neonatal white matter tract microstructure and 2-year language outcomes after preterm birth. NeuroImage: Clinical. 2020;28:102446. 10.1016/j.nicl.2020.102446. 33035964 PMC7554644

[59] Estrada KA, Govindaraj S, Abdi H, Moraglia LE, Wolff JJ, Meera SS, et al. Language exposure during infancy is negatively associated with white matter microstructure in the arcuate fasciculus. Developmental Cognitive Neuroscience. 2023;61:101240. 10.1016/j.dcn.2023.101240. 37060675 PMC10130606

[60] Chenausky K, Kernbach J, Norton A, Schlaug G White matter integrity and treatment-based change in speech performance in minimally verbal children with autism spectrum disorder. Front Hum Neurosci. 2017;11. 10.3389/fnhum.2017.00175 PMC5380725 28424605

[61] Andrews DS, Lee JK, Harvey DJ, Waizbard-Bartov E, Solomon M, Rogers SJ, et al. A longitudinal study of white matter development in relation to changes in autism severity across early childhood. Biological Psychiatry. 2021;89:424–32. 10.1016/j.biopsych.2020.10.013. 33349451 PMC7867569

[62] Surgent O, Andrews DS, Lee JK, Boyle J, Dakopolos A, Miller M, et al. Sex differences in the striatal contributions to longitudinal fine motor development in autistic children. Biological Psychiatry. 2025;S0006322325000277. 10.1016/j.biopsych.2025.01.005 PMC12124950 39818327

[63] Andrews DS, Lee JK, Solomon M, Rogers SJ, Amaral DG, Nordahl CW. A diffusion-weighted imaging tract-based spatial statistics study of autism spectrum disorder in preschool-aged children. J Neurodevelop Disord. 2019;11:32. 10.1186/s11689-019-9291-z. PMC6913008 31839001

[64] Vyshedskiy A, Pevzner A, Mack B, Shrayer E, Zea M, Bunner S, et al. Language comprehension developmental milestones in typically developing children assessed by the new language phenotype assessment (LPA). Children. 2025;12:793. 10.3390/children12060793. 40564751 PMC12191704

