# Supplementary material for: Multimodal predictors of spoken vocabulary development in autism: the role of early childhood brain and behavior
Source: Transl Psychiatry. 2026 Jun 19;16:436. doi: 10.1038/s41398-026-04168-2 (PMC13527143; doi:10.1038/s41398-026-04168-2)
Supplement: Supplementary file 1 [file 41398_2026_4168_MOESM1_ESM.docx]

**Supplementary Materials**

Multimodal Predictors of Spoken Vocabulary Development in Autism: The Role of Early Childhood Brain and Behavior

Olivia Surgent, Ph.D.^a^, Letitia Naigles, Ph.D.^b^, Andrew Dakopolos, Ph.D.^a^, Val K Sierakowski, BA^a^, Derek S Andrews, Ph.D.^a^, Joshua K Lee, Ph.D.^a^, Brianna Heath, Ph.D.^a^, Sally J Rogers, Ph.D.^a^, David G Amaral, Ph.D.^a^, Marjorie Solomon, Ph.D.^a^, Christine Wu Nordahl, Ph.D.^a^

^a^ MIND Institute and Department of Psychiatry and Behavioral Sciences, UC Davis School of Medicine, University of California Davis, Sacramento, CA, USA

^b^ Department of Psychological Sciences, University of Connecticut, Storrs, CT, USA

**Supplemental Methods**

*Scanner system upgrade.* All MRI scans were collected between 2007 and 2022. In August 2009, the Siemens 3T Trio was upgraded to a TIM system. While diffusion MRI parameters were not changed, post upgrade scans were associated with higher FA and AD values and lower MD and RD values. Thus, MRI system upgrade status was included as a covariate for all statistical analyses of diffusion indices.

**Table S1. Diffusion data quality comparisons**

|  | Starting Spoken Vocabulary Subgroups | | |  |  |
| --- | --- | --- | --- | --- | --- |
|  | Smaller  Mean (SD) | Average  Mean (SD) | Larger  Mean (SD) | F | *p* |
| Percent volumes with outliers | 12.55 (8.35) | 10.56 (8.54) | 11.78 (8.27) | 0.62 | .54 |
| Average slices per affected volume | 5.27 (2.76) | 5.43 (2.33) | 5.28 (2.69) | 0.02 | .98 |

**Supplemental Figures**

**Figure S1. Tractometry-based delineations of white matter pathways of interest.** The (A) inferior longitudinal fasciculus (ILF), (B) arcuate fasciculus (AF), (C) corticospinal tract (CST), and (D) uncinate fasciculus (UF) were each parcellated into three segments.

**Figure S2. Associations among FA and EOWPVT change rate by starting vocabulary group across white matter tracts of interest.** White matter tracts were parcellated into 9 segments and correlations were evaluated within each segment along the tracts. Orange boxes represent larger parcellated segments used in predictive brain + behavior analyses.

**Figure S3. Associations among behavioral measures and EOWPVT change rate across the full sample.**

**Figure S4. Associations among FA and EOWPVT change rate across the full sample.**

**Table S2. EOWPVT change rate predicted by fine motor skills in autistic children with smaller spoken vocabularies at Time 1**

| Predictor | b | Std. Error | t | p |
| --- | --- | --- | --- | --- |
| Fine Motor Skills (Time 1) | 0.09 | 0.03 | 2.72 | .01 |
| Age (Time 1) | -0.05 | 0.02 | -3.28 | .002 |
| EOWPVT raw score (Time 1) | 0.02 | 0.02 | 0.97 | .34 |
| EOWPVT, Expressive One Word Picture Vocabulary Test | | | | |

**Table S3. EOWPVT change rate predicted by fine motor skills and starting spoken vocabulary group**

| Predictor | Sum Sq | DF | F | *p* |
| --- | --- | --- | --- | --- |
| Intercept | 4.28 | 1 | 14.98 | <.001 |
| Fine Motor Skills (Time 1) | 0.21 | 1 | 0.74 | .39 |
| Vocabulary Subgroup | 1.82 | 2 | 3.18 | .05 |
| Age (Time 1) | 4.36 | 1 | 15.26 | <.001 |
| EOWPVT raw score (Time 1) | 0.10 | 1 | 0.34 | .56 |
| Fine Motor x Vocabulary Subgroup | 2.11 | 2 | 3.69 | .03 |
| EOWPVT, Expressive One Word Picture Vocabulary Test | | | | |

**Table S4. EOWPVT change rate predicted by joint attention skills in autistic children with smaller spoken vocabularies at Time 1**

| Predictor | b | Std. Error | t | p |
| --- | --- | --- | --- | --- |
| Joint Attention (Time 1) | -0.11 | 0.04 | -2.82 | .007 |
| Age (Time 1) | -0.03 | 0.01 | -2.04 | .04 |
| EOWPVT raw score (Time 1) | 0.02 | 0.02 | 1.24 | .22 |
| EOWPVT, Expressive One Word Picture Vocabulary Test | | | | |

**Table S5. EOWPVT change rate predicted by joint attention skills and starting spoken vocabulary group**

| Predictor | Sum Sq | DF | F | *p* |
| --- | --- | --- | --- | --- |
| Intercept | 4.89 | 1 | 17.22 | <.001 |
| Joint Attention (Time 1) | 0.22 | 1 | 0.78 | .38 |
| Vocabulary Subgroup | 0.42 | 2 | 0.74 | .48 |
| Age (Time 1) | 1.43 | 1 | 5.04 | .03 |
| EOWPVT raw score (Time 1) | 0.12 | 1 | 0.41 | .53 |
| Joint Attention x Vocabulary Subgroup | 0.71 | 2 | 1.26 | .29 |
| EOWPVT, Expressive One Word Picture Vocabulary Test | | | | |

**Table S6. Hierarchical regression model predicting EOWPVT change rate of autistic children in with smaller spoken vocabularies at Time 1**

| Predictor | Sum Sq | DF | F | *p* | Percent Variance |
| --- | --- | --- | --- | --- | --- |
| Intercept | 1.07 | 1 | 3.48 | 0.07 | 4.63 |
| Fine Motor Skills | 2.00 | 1 | 6.47 | 0.01 | 8.59 |
| Joint Attention | 1.97 | 1 | 6.36 | 0.01 | 8.46 |
| Age (months) | 2.43 | 1 | 7.87 | 0.007 | 10.46 |
| Time 1 EOWPVT raw score | 0.01 | 1 | 0.04 | 0.83 | 0.06 |
| EOWPVT, Expressive One Word Picture Vocabulary Test | | | | | |

**Table S7. EOWPVT change rate predicted by white matter microstructure of autistic children with smaller spoken vocabularies at Time 1**

| Metric | Tract | Segment | b | Std. Error | t | p | Adj p |
| --- | --- | --- | --- | --- | --- | --- | --- |
| FA | AF Left | Superior | -6.12 | 3.08 | -1.99 | .05 | .18 |
|  |  | **Central** | **-10.03** | **3.36** | **-2.98** | **.005** | **.05** |
|  |  | Inferior | -5.90 | 3.56 | -1.66 | .10 | .18 |
|  | AF Right | Superior | -9.20 | 4.80 | -1.92 | .06 | .18 |
|  |  | Central | -7.40 | 3.94 | -1.88 | .06 | .18 |
|  |  | Inferior | -5.72 | 2.83 | -2.02 | .05 | .18 |
|  | ILF Left | Temporal | -6.03 | 4.34 | -1.39 | .17 | .28 |
|  |  | Central | -5.71 | 3.21 | -1.78 | .08 | .18 |
|  |  | **Occipital** | **-12.84** | **3.67** | **-3.50** | **.001** | **.03** |
|  | ILF Right | Temporal | -6.84 | 4.06 | -1.68 | .10 | .18 |
|  |  | Central | -5.33 | 2.89 | -1.84 | .07 | .18 |
|  |  | **Occipital** | **-6.37** | **3.04** | **-2.10** | **.04** | **.18** |
|  | CST Left | **Inferior** | **-8.32** | **3.76** | **-2.21** | **.03** | **.18** |
|  |  | Central | -3.91 | 4.03 | -0.97 | .33 | .51 |
|  |  | Superior | -5.43 | 3.24 | -1.68 | .10 | .18 |
|  | CST Right | **Inferior** | **-9.02** | **4.17** | **-2.16** | **.03** | **.18** |
|  |  | Central | -2.04 | 3.14 | -0.65 | .52 | .69 |
|  |  | Superior | -0.79 | 3.24 | -0.24 | .80 | .98 |
|  | UF Left | Frontal | -1.08 | 3.55 | -0.30 | .76 | .98 |
|  |  | Central | -0.07 | 3.62 | -0.02 | .98 | .98 |
|  |  | Temporal | 0.28 | 3.55 | 0.08 | .93 | .89 |
|  | UF Right | Frontal | -2.63 | 4.97 | -0.53 | .59 | .89 |
|  |  | Central | -3.70 | 4.62 | -0.80 | .42 | .60 |
|  |  | Temporal | -0.95 | 3.97 | -0.24 | .81 | .76 |
| MD | AF Left | Superior | 1.83 | 2.70 | 0.68 | .50 | .97 |
|  |  | Central | 0.22 | 3.40 | 0.06 | .94 | .97 |
|  |  | Inferior | -2.89 | 3.23 | -0.90 | .37 | .97 |
|  | AF Right | Superior | 0.52 | 4.27 | 0.12 | .90 | .97 |
|  |  | Central | -0.80 | 3.49 | -0.23 | .82 | .97 |
|  |  | Inferior | 0.57 | 2.67 | 0.21 | .83 | .97 |
|  | ILF Left | Temporal | 1.29 | 2.75 | 0.47 | .64 | .97 |
|  |  | Central | 1.25 | 3.01 | 0.41 | .68 | .97 |
|  |  | Occipital | 2.53 | 3.49 | 0.72 | .47 | .97 |
|  | ILF Right | Temporal | -0.78 | 2.86 | -0.27 | .78 | .97 |
|  |  | Central | 1.22 | 2.54 | 0.48 | .63 | .97 |
|  |  | Occipital | 2.38 | 2.52 | 0.95 | .35 | .97 |
|  | CST Left | Inferior | 0.55 | 3.37 | 0.16 | .87 | .97 |
|  |  | Central | 0.83 | 2.34 | 0.36 | .72 | .97 |
|  |  | Superior | 0.65 | 1.16 | 0.56 | .57 | .97 |
|  | CST Right | Inferior | -7.28 | 3.73 | -1.95 | .05 | .97 |
|  |  | Central | -3.04 | 2.69 | -1.13 | .26 | .97 |
|  |  | Superior | 0.70 | 1.01 | 0.69 | .49 | .97 |
|  | UF Left | Frontal | -0.30 | 2.09 | -0.14 | .88 | .97 |
|  |  | Central | 0.50 | 2.76 | 0.18 | .85 | .97 |
|  |  | Temporal | -0.07 | 1.61 | -0.04 | .96 | .97 |
|  | UF Right | Frontal | 0.44 | 2.70 | 0.16 | .87 | .97 |
|  |  | Central | 0.46 | 1.86 | 0.24 | .80 | .97 |
|  |  | Temporal | -0.04 | 0.54 | -0.08 | .93 | .97 |
| AD | AF Left | Superior | -1.18 | 2.65 | -0.45 | .65 | .99 |
|  |  | Central | -4.74 | 3.09 | -1.53 | .13 | .79 |
|  |  | **Inferior** | **-8.15** | **3.12** | **-2.62** | **.01** | **.16** |
|  | AF Right | Superior | -3.78 | 3.60 | -1.05 | .30 | .82 |
|  |  | Central | -3.91 | 2.88 | -1.36 | .18 | .79 |
|  |  | Inferior | -3.11 | 2.37 | -1.31 | .19 | .79 |
|  | ILF Left | Temporal | 0.09 | 2.64 | 0.03 | .97 | .99 |
|  |  | Central | 0.61 | 2.19 | 0.28 | .78 | .99 |
|  |  | Occipital | -1.98 | 2.67 | -0.74 | .46 | .99 |
|  | ILF Right | Temporal | -2.28 | 2.41 | -0.95 | .35 | .84 |
|  |  | Central | -0.14 | 1.56 | -0.09 | .92 | .99 |
|  |  | Occipital | -0.20 | 1.95 | -0.10 | .92 | .99 |
|  | CST Left | Inferior | -2.49 | 2.39 | -1.04 | .30 | .99 |
|  |  | Central | 0.06 | 1.60 | 0.04 | .97 | .99 |
|  |  | Superior | 0.10 | 0.88 | 0.11 | .90 | .82 |
|  | CST Right | **Inferior** | **-9.21** | **2.30** | **-4.01** | **.0003** | **.01** |
|  |  | Central | -2.84 | 2.05 | -1.38 | .17 | .79 |
|  |  | Superior | 0.99 | 0.85 | 1.16 | .25 | .82 |
|  | UF Left | Frontal | 0.02 | 2.05 | 0.01 | .99 | .99 |
|  |  | Central | 1.42 | 2.73 | 0.52 | .60 | .99 |
|  |  | Temporal | 0.02 | 1.34 | 0.02 | .98 | .99 |
|  | UF Right | Frontal | -0.70 | 2.93 | -0.24 | .81 | .99 |
|  |  | Central | 0.24 | 1.50 | 0.16 | .87 | .99 |
|  |  | Temporal | -0.04 | 0.37 | -0.11 | .90 | .99 |
| RD | AF Left | Superior | 2.31 | 2.49 | 0.92 | .36 | .98 |
|  |  | Central | 3.22 | 2.99 | 1.08 | .28 | .98 |
|  |  | Inferior | -0.19 | 2.96 | -0.06 | .95 | .98 |
|  | AF Right | Superior | 2.04 | 3.79 | 0.54 | .59 | .98 |
|  |  | Central | 1.76 | 3.32 | 0.53 | .60 | .98 |
|  |  | Inferior | 1.84 | 2.48 | 0.74 | .46 | .98 |
|  | ILF Left | Temporal | 1.89 | 2.75 | 0.69 | .49 | .98 |
|  |  | Central | 2.99 | 3.27 | 0.92 | .36 | .98 |
|  |  | Occipital | 6.15 | 3.62 | 1.70 | .09 | .98 |
|  | ILF Right | Temporal | 0.69 | 2.84 | 0.24 | .81 | .98 |
|  |  | Central | 3.47 | 2.81 | 1.23 | .22 | .98 |
|  |  | Occipital | 4.02 | 2.60 | 1.55 | .13 | .98 |
|  | CST Left | Inferior | 2.91 | 3.19 | 0.91 | .36 | .98 |
|  |  | Central | 0.51 | 2.56 | 0.20 | .84 | .98 |
|  |  | Superior | 0.79 | 1.24 | 0.64 | .52 | .98 |
|  | CST Right | Inferior | -1.40 | 3.44 | -0.41 | .68 | .98 |
|  |  | Central | -0.93 | 2.15 | -0.43 | .66 | .98 |
|  |  | Superior | 0.48 | 1.04 | 0.46 | .64 | .98 |
|  | UF Left | Frontal | -0.07 | 1.90 | -0.04 | .96 | .98 |
|  |  | Central | 0.06 | 2.48 | 0.02 | .98 | .98 |
|  |  | Temporal | -0.31 | 1.72 | -0.18 | .85 | .98 |
|  | UF Right | Frontal | 0.43 | 2.60 | 0.16 | .87 | .98 |
|  |  | Central | 0.64 | 2.00 | 0.32 | .75 | .98 |
|  |  | Temporal | -0.06 | 0.67 | -0.08 | .93 | .98 |
| All analyses additionally controlled for Time 1 age, Time 1 EOWPVT raw score, sex assigned at birth, average head motion and scanner system  FA, Fractional Anisotropy; MD, Mean Diffusivity; AD, Axial Diffusivity; RD, Radial Diffusivity, AF, Arcuate Fasciculus; ILF, Inferior Longitudinal Fasciculus; CST, Corticospinal Tract; UF, uncinate fasciculus; EOWPVT, Expressive One Word Picture Vocabulary Test | | | | | | | |

**Table S8. EOWPVT change rate predicted by white matter FA and starting spoken vocabulary group**

| Projection | Segment | Predictor | b | Std. Error | t | p | Adj p |
| --- | --- | --- | --- | --- | --- | --- | --- |
| AF Left | Superior | FA | 2.62 | 1.60 | 1.64 | .105 | .419 |
|  |  | Vocabulary Subgroup | -0.07 | 0.17 | -0.38 | .701 | .973 |
|  |  | **FA x Group** | **-8.89** | **3.09** | **-2.87** | **.005** | **.024** |
|  | Central | FA | 2.72 | 1.76 | 1.54 | .126 | .419 |
|  |  | Vocabulary Subgroup | -0.07 | 0.17 | -0.44 | .663 | .973 |
|  |  | **FA x Group** | **-12.83** | **3.49** | **-3.68** | **<.001** | **.005** |
|  | Inferior | FA | 4.47 | 2.01 | 2.22 | .028 | .389 |
|  |  | Vocabulary Subgroup | -0.04 | 0.17 | -0.24 | .809 | .973 |
|  |  | **FA x Group** | **-10.64** | **3.61** | **-2.95** | **.004** | **.024** |
| AF Right | Superior | FA | 3.32 | 2.38 | 1.40 | .165 | .419 |
|  |  | Vocabulary Subgroup | -0.08 | 0.17 | -0.48 | .636 | .973 |
|  |  | **FA x Group** | **-12.89** | **4.73** | **-2.73** | **.008** | **.030** |
|  | Central | FA | 2.70 | 1.98 | 1.37 | .174 | .419 |
|  |  | Vocabulary Subgroup | -0.03 | 0.17 | -0.20 | .844 | .973 |
|  |  | **FA x Group** | **-9.98** | **3.92** | **-2.55** | **.012** | **.042** |
|  | Inferior | FA | 3.22 | 1.61 | 2.00 | .049 | .389 |
|  |  | Vocabulary Subgroup | -0.06 | 0.17 | -0.34 | .737 | .973 |
|  |  | **FA x Group** | **-9.04** | **2.91** | **-3.11** | **.002** | **.019** |
| ILF Left | Temporal | FA | 0.22 | 1.57 | 0.14 | .887 | .952 |
|  |  | Vocabulary Subgroup | -0.06 | 0.18 | -0.32 | .750 | .973 |
|  |  | FA x Group | -5.79 | 4.01 | -1.44 | .152 | .214 |
|  | Central | FA | 1.90 | 1.30 | 1.46 | .146 | .419 |
|  |  | Vocabulary Subgroup | -0.04 | 0.17 | -0.26 | .799 | .973 |
|  |  | FA x Group | -6.82 | 2.99 | -2.28 | .025 | .065 |
|  | Occipital | FA | 1.57 | 1.33 | 1.18 | .241 | .526 |
|  |  | Vocabulary Subgroup | -0.11 | 0.17 | -0.65 | .519 | .973 |
|  |  | **FA x Group** | **-13.69** | **3.60** | **-3.81** | **<.001** | **.005** |
| ILF Right | Temporal | FA | 0.22 | 1.77 | 0.12 | .902 | .952 |
|  |  | Vocabulary Subgroup | -0.05 | 0.17 | -0.32 | .751 | .973 |
|  |  | FA x Group | -7.20 | 3.84 | -1.87 | .064 | .117 |
|  | Central | FA | 0.48 | 1.46 | 0.33 | .741 | .952 |
|  |  | Vocabulary Subgroup | -0.01 | 0.17 | -0.08 | .939 | .973 |
|  |  | FA x Group | -5.77 | 2.89 | -1.99 | .049 | .098 |
|  | Occipital | FA | 0.10 | 1.73 | 0.06 | .952 | .952 |
|  |  | Vocabulary Subgroup | -0.04 | 0.17 | -0.23 | .819 | .973 |
|  |  | FA x Group | -6.46 | 3.16 | -2.05 | .043 | .094 |
| CST Left | Inferior | FA | 0.26 | 1.32 | 0.19 | .846 | .952 |
|  |  | Vocabulary Subgroup | -0.06 | 0.17 | -0.37 | .710 | .973 |
|  |  | **FA x Group** | **-8.62** | **3.55** | **-2.43** | **.017** | **.051** |
|  | Central | FA | -0.18 | 0.99 | -0.18 | .857 | .952 |
|  |  | Vocabulary Subgroup | -0.02 | 0.18 | -0.10 | .917 | .973 |
|  |  | FA x Group | -3.79 | 3.54 | -1.07 | .287 | .382 |
|  | Superior | FA | 0.16 | 1.00 | 0.16 | .873 | .952 |
|  |  | Vocabulary Subgroup | -0.01 | 0.17 | -0.03 | .973 | .973 |
|  |  | FA x Group | -4.90 | 2.92 | -1.68 | .096 | .154 |
| CST Right | Inferior | FA | -0.17 | 2.27 | -0.08 | .939 | .952 |
|  |  | Vocabulary Subgroup | -0.08 | 0.17 | -0.47 | .642 | .973 |
|  |  | FA x Group | -9.10 | 4.26 | -2.14 | .035 | .084 |
|  | Central | FA | -0.62 | 1.17 | -0.53 | .600 | .952 |
|  |  | Vocabulary Subgroup | -0.02 | 0.18 | -0.13 | .894 | .973 |
|  |  | FA x Group | -1.36 | 2.88 | -0.47 | .637 | .665 |
|  | Superior | FA | 0.45 | 1.18 | 0.38 | .703 | .952 |
|  |  | Vocabulary Subgroup | -0.03 | 0.18 | -0.20 | .844 | .973 |
|  |  | FA x Group | -0.97 | 2.95 | -0.33 | .744 | .744 |
| UF Left | Frontal | FA | 1.15 | 1.94 | 0.59 | .554 | .949 |
|  |  | Vocabulary Subgroup | -0.04 | 0.18 | -0.23 | .816 | .973 |
|  |  | FA x Group | -2.49 | 3.52 | -0.71 | .481 | .578 |
|  | Central | FA | 1.07 | 1.69 | 0.64 | .526 | .949 |
|  |  | Vocabulary Subgroup | -0.04 | 0.18 | -0.24 | .810 | .973 |
|  |  | FA x Group | -1.65 | 3.42 | -0.48 | .631 | .665 |
|  | Temporal | FA | 2.34 | 1.55 | 1.50 | .136 | .419 |
|  |  | Vocabulary Subgroup | -0.06 | 0.18 | -0.37 | .715 | .973 |
|  |  | FA x Group | -2.16 | 3.34 | -0.65 | .519 | .593 |
| UF Right | Frontal | FA | 2.50 | 2.37 | 1.06 | .294 | .588 |
|  |  | Vocabulary Subgroup | -0.05 | 0.18 | -0.29 | .773 | .973 |
|  |  | FA x Group | -4.64 | 4.74 | -0.98 | .330 | .417 |
|  | Central | FA | 3.14 | 1.85 | 1.70 | .093 | .419 |
|  |  | Vocabulary Subgroup | -0.05 | 0.17 | -0.29 | .776 | .973 |
|  |  | FA x Group | -7.41 | 4.17 | -1.77 | .079 | .135 |
|  | Temporal | FA | 3.84 | 1.81 | 2.12 | .037 | .389 |
|  |  | Vocabulary Subgroup | -0.01 | 0.17 | -0.09 | .932 | .973 |
|  |  | FA x Group | -5.09 | 3.51 | -1.45 | .150 | .214 |
| All analyses additionally controlled for Time 1 age, Time 1 EOWPVT raw score, sex assigned at birth, average head motion and scanner system  FA, Fractional Anisotropy; Arcuate Fasciculus; ILF, Inferior Longitudinal Fasciculus; CST, Corticospinal Tract; UF, uncinate fasciculus; EOWPVT, Expressive One Word Picture Vocabulary Test | | | | | | | |

**Table S9. Pairwise comparisons of simple slopes in regions with significant FA-by-start group interaction effects**

| Region | Contrast | Δ Slope | SE | t | p |
| --- | --- | --- | --- | --- | --- |
| Left Superior AF | Smaller - Average | -9.29 | 3.91 | -2.38 | .05 |
|  | Smaller - Larger | -8.73 | 3.30 | -2.64 | .02 |
|  | Average - Larger | 0.56 | 3.43 | 0.16 | .98 |
| Left Central AF | Smaller - Average | -14.46 | 4.14 | -3.49 | .002 |
|  | Smaller - Larger | -11.80 | 3.78 | -3.12 | .006 |
|  | Average - Larger | 2.67 | 3.59 | 0.74 | .73 |
| Left Inferior AF | Smaller - Average | -12.58 | 4.05 | -3.11 | .007 |
|  | Smaller - Larger | -8.28 | 4.29 | -1.93 | .13 |
|  | Average - Larger | 4.30 | 4.07 | 1.06 | .54 |
| Right Superior AF | Smaller - Average | -12.19 | 5.48 | -2.55 | .03 |
|  | Smaller - Larger | -13.48 | 5.28 | -2.33 | .07 |
|  | Average - Larger | -1.29 | 4.93 | -0.26 | .96 |
| Right Central AF | Smaller - Average | -10.95 | 4.93 | -2.22 | .07 |
|  | Smaller - Larger | -9.47 | 4.23 | -2.24 | .07 |
|  | Average - Larger | 1.48 | 4.45 | 0.33 | .94 |
| Right Inferior AF | Smaller - Average | -9.85 | 3.72 | -2.65 | .02 |
|  | Smaller - Larger | -8.68 | 3.15 | -2.75 | .01 |
|  | Average - Larger | 1.16 | 3.44 | 0.34 | .93 |
| Left Occipital ILF | Smaller - Average | -12.20 | 4.42 | -2.77 | .01 |
|  | Smaller - Larger | -14.20 | 3.72 | -3.83 | <.001 |
|  | Average - Larger | -2.00 | 3.33 | -0.60 | .81 |
| Left Inferior CST | Smaller - Average | -7.53 | 3.78 | -1.99 | .11 |
|  | Smaller - Larger | -9.97 | 3.87 | -2.58 | .03 |
|  | Average - Larger | -2.44 | 2.69 | -0.91 | .63 |

**Table S10. EOWPVT change rate predicted by white matter MD and starting spoken vocabulary group**

| Projection | Segment | Predictor | b | Std. Error | t | p | Adj p |
| --- | --- | --- | --- | --- | --- | --- | --- |
| AF Left | Superior | MD | -2.50 | 2.35 | -1.07 | .289 | .602 |
|  |  | Vocabulary Subgroup | -0.02 | 0.18 | -0.14 | .888 | .992 |
|  |  | MD x Group | 4.22 | 3.26 | 1.30 | .198 | .717 |
|  | Central | MD | -5.06 | 3.07 | -1.65 | .102 | .602 |
|  |  | Vocabulary Subgroup | 0.00 | 0.18 | -0.03 | .979 | .992 |
|  |  | MD x Group | 5.20 | 4.17 | 1.25 | .215 | .717 |
|  | Inferior | MD | -2.22 | 2.78 | -0.80 | .426 | .602 |
|  |  | Vocabulary Subgroup | -0.01 | 0.18 | -0.06 | .956 | .992 |
|  |  | MD x Group | -0.82 | 3.90 | -0.21 | .835 | .835 |
| AF Right | Superior | MD | -3.38 | 2.95 | -1.15 | .254 | .602 |
|  |  | Vocabulary Subgroup | 0.00 | 0.18 | -0.02 | .985 | .992 |
|  |  | MD x Group | 4.16 | 4.60 | 0.90 | .369 | .717 |
|  | Central | MD | -3.00 | 2.73 | -1.10 | .275 | .602 |
|  |  | Vocabulary Subgroup | -0.01 | 0.18 | -0.08 | .940 | .992 |
|  |  | MD x Group | 2.12 | 3.99 | 0.53 | .598 | .717 |
|  | Inferior | MD | -1.72 | 2.45 | -0.70 | .484 | .611 |
|  |  | Vocabulary Subgroup | -0.02 | 0.18 | -0.10 | .920 | .992 |
|  |  | MD x Group | 2.09 | 3.31 | 0.63 | .530 | .717 |
| ILF Left | Temporal | MD | -1.28 | 2.38 | -0.54 | .594 | .678 |
|  |  | Vocabulary Subgroup | -0.04 | 0.18 | -0.25 | .806 | .992 |
|  |  | MD x Group | 2.49 | 3.33 | 0.75 | .456 | .717 |
|  | Central | MD | -2.37 | 2.19 | -1.08 | .282 | .602 |
|  |  | Vocabulary Subgroup | -0.02 | 0.18 | -0.14 | .892 | .992 |
|  |  | MD x Group | 3.47 | 3.34 | 1.04 | .301 | .717 |
|  | Occipital | MD | -3.48 | 2.74 | -1.27 | .207 | .602 |
|  |  | Vocabulary Subgroup | -0.02 | 0.17 | -0.09 | .927 | .992 |
|  |  | MD x Group | 6.11 | 4.02 | 1.52 | .131 | .717 |
| ILF Right | Temporal | MD | -1.93 | 2.48 | -0.78 | .438 | .602 |
|  |  | Vocabulary Subgroup | -0.03 | 0.18 | -0.15 | .879 | .992 |
|  |  | MD x Group | 0.93 | 3.42 | 0.27 | .785 | .820 |
|  | Central | MD | -3.51 | 2.14 | -1.64 | .105 | .602 |
|  |  | Vocabulary Subgroup | -0.01 | 0.17 | -0.08 | .933 | .992 |
|  |  | MD x Group | 4.67 | 3.02 | 1.55 | .124 | .717 |
|  | Occipital | MD | -2.10 | 2.41 | -0.87 | .386 | .602 |
|  |  | Vocabulary Subgroup | -0.01 | 0.18 | -0.07 | .942 | .992 |
|  |  | MD x Group | 4.48 | 3.22 | 1.39 | .166 | .717 |
| CST Left | Inferior | MD | -1.88 | 1.67 | -1.12 | .264 | .602 |
|  |  | Vocabulary Subgroup | -0.04 | 0.18 | -0.25 | .804 | .992 |
|  |  | MD x Group | 2.37 | 3.27 | 0.72 | .471 | .717 |
|  | Central | MD | -1.01 | 1.33 | -0.76 | .451 | .602 |
|  |  | Vocabulary Subgroup | -0.03 | 0.18 | -0.17 | .863 | .992 |
|  |  | MD x Group | 1.69 | 2.32 | 0.73 | .468 | .717 |
|  | Superior | MD | -0.51 | 0.50 | -1.03 | .307 | .602 |
|  |  | Vocabulary Subgroup | 0.00 | 0.18 | 0.01 | .992 | .992 |
|  |  | MD x Group | 0.93 | 1.03 | 0.90 | .370 | .717 |
| CST Right | Inferior | MD | -1.77 | 1.77 | -1.00 | .320 | .602 |
|  |  | Vocabulary Subgroup | 0.00 | 0.17 | -0.01 | .990 | .992 |
|  |  | MD x Group | -5.09 | 3.67 | -1.39 | .169 | .717 |
|  | Central | MD | -1.07 | 1.89 | -0.56 | .574 | .678 |
|  |  | Vocabulary Subgroup | -0.04 | 0.17 | -0.22 | .829 | .992 |
|  |  | MD x Group | -1.76 | 2.88 | -0.61 | .543 | .717 |
|  | Superior | MD | 0.05 | 0.28 | 0.16 | .870 | .870 |
|  |  | Vocabulary Subgroup | -0.03 | 0.18 | -0.15 | .880 | .992 |
|  |  | MD x Group | 0.61 | 0.88 | 0.69 | .489 | .717 |
| UF Left | Frontal | MD | -1.69 | 1.53 | -1.11 | .271 | .602 |
|  |  | Vocabulary Subgroup | -0.05 | 0.18 | -0.29 | .773 | .992 |
|  |  | MD x Group | 1.31 | 2.29 | 0.57 | .570 | .717 |
|  | Central | MD | -1.29 | 1.54 | -0.84 | .403 | .602 |
|  |  | Vocabulary Subgroup | -0.06 | 0.18 | -0.32 | .750 | .992 |
|  |  | MD x Group | 1.69 | 2.76 | 0.61 | .543 | .717 |
|  | Temporal | MD | 0.64 | 1.44 | 0.45 | .656 | .715 |
|  |  | Vocabulary Subgroup | -0.03 | 0.18 | -0.18 | .854 | .992 |
|  |  | MD x Group | -0.85 | 1.95 | -0.43 | .665 | .760 |
| UF Right | Frontal | MD | -0.77 | 2.64 | -0.29 | .770 | .804 |
|  |  | Vocabulary Subgroup | -0.03 | 0.18 | -0.18 | .860 | .992 |
|  |  | MD x Group | 0.95 | 3.45 | 0.28 | .783 | .820 |
|  | Central | MD | -2.13 | 1.25 | -1.70 | .092 | .602 |
|  |  | Vocabulary Subgroup | -0.05 | 0.17 | -0.30 | .767 | .992 |
|  |  | MD x Group | 2.49 | 1.96 | 1.27 | .206 | .717 |
|  | Temporal | MD | -1.65 | 1.77 | -0.93 | .353 | .602 |
|  |  | Vocabulary Subgroup | 0.00 | 0.18 | 0.02 | .987 | .992 |
|  |  | MD x Group | 1.58 | 1.84 | 0.86 | .391 | .717 |
| All analyses additionally controlled for Time 1 age, Time 1 EOWPVT scores, sex assigned at birth, average head motion and scanner system  MD, Mean Diffusivity; Arcuate Fasciculus; ILF, Inferior Longitudinal Fasciculus; CST, Corticospinal Tract; UF, uncinate fasciculus; EOWPVT, Expressive One Word Picture Vocabulary Test | | | | | | | |

**Table S11. EOWPVT change rate predicted by white matter AD and starting spoken Vocabulary Subgroup**

| Projection | Segment | Predictor | b | Std. Error | t | p | Adj p |
| --- | --- | --- | --- | --- | --- | --- | --- |
| AF Left | Superior | AD | 0.72 | 2.13 | 0.34 | .737 | .877 |
|  |  | Vocabulary Subgroup | -0.03 | 0.18 | -0.20 | .843 | .994 |
|  |  | AD x Group | -2.03 | 3.02 | -0.67 | .504 | .860 |
|  | Central | AD | -2.09 | 2.25 | -0.93 | .353 | .877 |
|  |  | Vocabulary Subgroup | -0.05 | 0.17 | -0.30 | .765 | .994 |
|  |  | AD x Group | -2.70 | 3.42 | -0.79 | .432 | .860 |
|  | Inferior | AD | 1.02 | 2.08 | 0.49 | .623 | .877 |
|  |  | Vocabulary Subgroup | -0.03 | 0.17 | -0.16 | .877 | .994 |
|  |  | AD x Group | **-9.36** | **3.43** | **-2.73** | **.008** | **.180** |
| AF Right | Superior | AD | -0.89 | 2.02 | -0.44 | .661 | .877 |
|  |  | Vocabulary Subgroup | -0.03 | 0.18 | -0.15 | .881 | .994 |
|  |  | AD x Group | -2.49 | 3.59 | -0.69 | .489 | .860 |
|  | Central | AD | -1.37 | 1.66 | -0.83 | .410 | .877 |
|  |  | Vocabulary Subgroup | -0.06 | 0.17 | -0.33 | .739 | .994 |
|  |  | AD x Group | -2.36 | 2.94 | -0.80 | .424 | .860 |
|  | Inferior | AD | 1.07 | 1.67 | 0.64 | .522 | .877 |
|  |  | Vocabulary Subgroup | -0.07 | 0.18 | -0.39 | .701 | .994 |
|  |  | AD x Group | -4.12 | 2.56 | -1.61 | .110 | .744 |
| ILF Left | Temporal | AD | 0.12 | 1.34 | 0.09 | .931 | .931 |
|  |  | Vocabulary Subgroup | -0.03 | 0.18 | -0.19 | .850 | .994 |
|  |  | AD x Group | 0.04 | 2.60 | 0.01 | .988 | .988 |
|  | Central | AD | 0.13 | 1.06 | 0.12 | .902 | .931 |
|  |  | Vocabulary Subgroup | -0.04 | 0.18 | -0.20 | .841 | .994 |
|  |  | AD x Group | 0.62 | 2.10 | 0.30 | .767 | .877 |
|  | Occipital | AD | 0.75 | 1.32 | 0.57 | .570 | .877 |
|  |  | Vocabulary Subgroup | -0.03 | 0.18 | -0.19 | .848 | .994 |
|  |  | AD x Group | -2.48 | 2.61 | -0.95 | .344 | .860 |
| ILF Right | Temporal | AD | -1.02 | 1.19 | -0.86 | .394 | .877 |
|  |  | Vocabulary Subgroup | -0.06 | 0.18 | -0.31 | .754 | .994 |
|  |  | AD x Group | -1.33 | 2.25 | -0.59 | .557 | .860 |
|  | Central | AD | -1.09 | 0.85 | -1.29 | .200 | .799 |
|  |  | Vocabulary Subgroup | -0.04 | 0.17 | -0.20 | .841 | .994 |
|  |  | AD x Group | 1.04 | 1.55 | 0.67 | .503 | .860 |
|  | Occipital | AD | -0.54 | 1.14 | -0.47 | .639 | .877 |
|  |  | Vocabulary Subgroup | -0.03 | 0.18 | -0.19 | .850 | .994 |
|  |  | AD x Group | 0.35 | 2.00 | 0.18 | .860 | .897 |
| CST Left | Inferior | AD | -1.88 | 1.29 | -1.45 | .150 | .799 |
|  |  | Vocabulary Subgroup | -0.05 | 0.17 | -0.27 | .786 | .994 |
|  |  | AD x Group | -0.55 | 2.38 | -0.23 | .818 | .893 |
|  | Central | AD | -2.54 | 1.10 | -2.30 | .023 | .563 |
|  |  | Vocabulary Subgroup | 0.01 | 0.17 | 0.04 | .966 | .994 |
|  |  | AD x Group | 2.62 | 1.69 | 1.55 | .124 | .744 |
|  | Superior | AD | -0.48 | 0.48 | -1.02 | .312 | .877 |
|  |  | Vocabulary Subgroup | 0.00 | 0.18 | -0.01 | .994 | .994 |
|  |  | AD x Group | 0.53 | 0.83 | 0.63 | .528 | .860 |
| CST Right | Inferior | AD | -2.53 | 1.38 | -1.83 | .070 | .799 |
|  |  | Vocabulary Subgroup | -0.03 | 0.16 | -0.18 | .855 | .994 |
|  |  | AD x Group | **-6.18** | **2.54** | **-2.43** | **.017** | **.201** |
|  | Central | AD | -1.55 | 1.07 | -1.44 | .153 | .799 |
|  |  | Vocabulary Subgroup | -0.01 | 0.17 | -0.07 | .944 | .994 |
|  |  | AD x Group | -1.04 | 1.99 | -0.52 | .601 | .860 |
|  | Superior | AD | 0.03 | 0.26 | 0.14 | .893 | .931 |
|  |  | Vocabulary Subgroup | -0.02 | 0.18 | -0.09 | .926 | .994 |
|  |  | AD x Group | 0.93 | 0.75 | 1.24 | .218 | .860 |
| UF Left | Frontal | AD | -0.82 | 1.23 | -0.67 | .507 | .877 |
|  |  | Vocabulary Subgroup | -0.05 | 0.18 | -0.27 | .788 | .994 |
|  |  | AD x Group | 0.70 | 2.05 | 0.34 | .733 | .877 |
|  | Central | AD | -0.46 | 1.53 | -0.30 | .767 | .877 |
|  |  | Vocabulary Subgroup | -0.04 | 0.18 | -0.20 | .842 | .994 |
|  |  | AD x Group | 1.36 | 2.66 | 0.51 | .609 | .860 |
|  | Temporal | AD | 1.17 | 0.87 | 1.35 | .181 | .799 |
|  |  | Vocabulary Subgroup | -0.04 | 0.17 | -0.21 | .835 | .994 |
|  |  | AD x Group | -1.25 | 1.39 | -0.90 | .370 | .860 |
| UF Right | Frontal | AD | 0.50 | 1.50 | 0.33 | .742 | .877 |
|  |  | Vocabulary Subgroup | -0.04 | 0.18 | -0.21 | .836 | .994 |
|  |  | AD x Group | -1.19 | 2.83 | -0.42 | .675 | .877 |
|  | Central | AD | -1.34 | 1.19 | -1.13 | .262 | .877 |
|  |  | Vocabulary Subgroup | -0.05 | 0.18 | -0.26 | .798 | .994 |
|  |  | AD x Group | 1.38 | 1.68 | 0.82 | .412 | .860 |
|  | Temporal | AD | 0.33 | 1.10 | 0.30 | .766 | .877 |
|  |  | Vocabulary Subgroup | -0.04 | 0.18 | -0.23 | .820 | .994 |
|  |  | AD x Group | -0.40 | 1.14 | -0.35 | .726 | .877 |
| All analyses additionally controlled for Time 1 age, Time 1 EOWPVT raw score, sex, average head motion and scanner system  AD, Axial Diffusivity; Arcuate Fasciculus; ILF, Inferior Longitudinal Fasciculus; CST, Corticospinal Tract; UF, uncinate fasciculus; EOWPVT, Expressive One Word Picture Vocabulary Test | | | | | | | |

**Table S12. EOWPVT change rate predicted by white matter RD and starting spoken vocabulary group**

| Projection | Segment | Predictor | b | Std. Error | t | p | Adj p |
| --- | --- | --- | --- | --- | --- | --- | --- |
| AF Left | Superior | RD | -2.55 | 1.84 | -1.39 | .168 | .406 |
|  |  | Vocabulary Subgroup | -0.03 | 0.17 | -0.17 | .868 | .998 |
|  |  | RD x Group | 4.85 | 2.79 | 1.74 | .085 | .341 |
|  | Central | RD | -4.66 | 2.33 | -2.00 | .048 | .406 |
|  |  | Vocabulary Subgroup | 0.00 | 0.17 | 0.00 | .998 | .998 |
|  |  | RD x Group | **7.84** | **3.42** | **2.29** | **.024** | **.287** |
|  | Inferior | RD | -3.39 | 2.32 | -1.46 | .146 | .406 |
|  |  | Vocabulary Subgroup | 0.00 | 0.18 | 0.01 | .996 | .998 |
|  |  | RD x Group | 3.16 | 3.39 | 0.93 | .354 | .567 |
| AF Right | Superior | RD | -3.95 | 2.75 | -1.44 | .154 | .406 |
|  |  | Vocabulary Subgroup | 0.00 | 0.18 | 0.00 | .997 | .998 |
|  |  | RD x Group | 6.18 | 4.19 | 1.47 | .143 | .383 |
|  | Central | RD | -3.36 | 2.43 | -1.38 | .169 | .406 |
|  |  | Vocabulary Subgroup | 0.00 | 0.18 | 0.03 | .980 | .998 |
|  |  | RD x Group | 4.95 | 3.69 | 1.34 | .182 | .397 |
|  | Inferior | RD | -2.99 | 2.10 | -1.42 | .158 | .406 |
|  |  | Vocabulary Subgroup | -0.01 | 0.17 | -0.06 | .956 | .998 |
|  |  | RD x Group | 4.66 | 2.95 | 1.58 | .117 | .352 |
| ILF Left | Temporal | RD | -0.99 | 1.32 | -0.75 | .455 | .642 |
|  |  | Vocabulary Subgroup | -0.05 | 0.18 | -0.31 | .755 | .998 |
|  |  | RD x Group | 3.82 | 2.99 | 1.28 | .204 | .409 |
|  | Central | RD | -0.12 | 0.96 | -0.13 | .898 | .957 |
|  |  | Vocabulary Subgroup | -0.03 | 0.18 | -0.18 | .856 | .998 |
|  |  | RD x Group | 0.60 | 2.33 | 0.26 | .797 | .869 |
|  | Occipital | RD | -0.49 | 0.49 | -1.01 | .314 | .503 |
|  |  | Vocabulary Subgroup | 0.00 | 0.18 | 0.00 | .997 | .998 |
|  |  | RD x Group | 1.02 | 1.08 | 0.94 | .348 | .567 |
| ILF Right | Temporal | RD | -0.81 | 1.74 | -0.47 | .641 | .758 |
|  |  | Vocabulary Subgroup | -0.03 | 0.18 | -0.16 | .874 | .998 |
|  |  | RD x Group | -0.25 | 3.37 | -0.07 | .941 | .941 |
|  | Central | RD | 0.06 | 1.26 | 0.05 | .960 | .960 |
|  |  | Vocabulary Subgroup | -0.03 | 0.18 | -0.19 | .847 | .998 |
|  |  | RD x Group | -1.02 | 2.15 | -0.47 | .636 | .742 |
|  | Occipital | RD | 0.03 | 0.28 | 0.10 | .917 | .957 |
|  |  | Vocabulary Subgroup | -0.03 | 0.18 | -0.17 | .862 | .998 |
|  |  | RD x Group | 0.41 | 0.90 | 0.46 | .649 | .742 |
| CST Left | Inferior | RD | -1.28 | 2.12 | -0.60 | .549 | .701 |
|  |  | Vocabulary Subgroup | -0.04 | 0.18 | -0.24 | .812 | .998 |
|  |  | RD x Group | 2.95 | 3.14 | 0.94 | .350 | .567 |
|  | Central | RD | -2.79 | 1.79 | -1.56 | .121 | .406 |
|  |  | Vocabulary Subgroup | -0.03 | 0.17 | -0.16 | .871 | .998 |
|  |  | RD x Group | 5.40 | 3.25 | 1.66 | .099 | .341 |
|  | Superior | RD | -3.09 | 2.05 | -1.51 | .134 | .406 |
|  |  | Vocabulary Subgroup | -0.02 | 0.17 | -0.13 | .893 | .998 |
|  |  | RD x Group | **9.02** | **3.70** | **2.44** | **.017** | **.287** |
| CST Right | Inferior | RD | -1.55 | 2.63 | -0.59 | .555 | .701 |
|  |  | Vocabulary Subgroup | -0.03 | 0.18 | -0.16 | .874 | .998 |
|  |  | RD x Group | 1.90 | 3.53 | 0.54 | .590 | .742 |
|  | Central | RD | -2.61 | 2.27 | -1.15 | .254 | .469 |
|  |  | Vocabulary Subgroup | -0.01 | 0.17 | -0.05 | .963 | .998 |
|  |  | RD x Group | 5.82 | 3.29 | 1.77 | .080 | .341 |
|  | Superior | RD | -2.36 | 2.54 | -0.93 | .356 | .534 |
|  |  | Vocabulary Subgroup | -0.02 | 0.17 | -0.11 | .914 | .998 |
|  |  | RD x Group | 6.39 | 3.38 | 1.89 | .061 | .341 |
| UF Left | Frontal | RD | -1.64 | 1.37 | -1.20 | .234 | .469 |
|  |  | Vocabulary Subgroup | -0.05 | 0.18 | -0.28 | .776 | .998 |
|  |  | RD x Group | 1.49 | 2.08 | 0.72 | .476 | .672 |
|  | Central | RD | -1.57 | 1.34 | -1.17 | .246 | .469 |
|  |  | Vocabulary Subgroup | -0.06 | 0.18 | -0.33 | .743 | .998 |
|  |  | RD x Group | 1.61 | 2.47 | 0.65 | .515 | .687 |
|  | Temporal | RD | -0.76 | 1.75 | -0.44 | .663 | .758 |
|  |  | Vocabulary Subgroup | -0.04 | 0.18 | -0.20 | .842 | .998 |
|  |  | RD x Group | 0.33 | 2.25 | 0.15 | .884 | .923 |
| UF Right | Frontal | RD | -2.88 | 2.77 | -1.04 | .302 | .503 |
|  |  | Vocabulary Subgroup | -0.03 | 0.18 | -0.19 | .848 | .998 |
|  |  | RD x Group | 3.00 | 3.50 | 0.86 | .394 | .591 |
|  | Central | RD | -2.16 | 1.15 | -1.87 | .064 | .406 |
|  |  | Vocabulary Subgroup | -0.05 | 0.17 | -0.30 | .763 | .998 |
|  |  | RD x Group | 2.73 | 1.99 | 1.37 | .172 | .397 |
|  | Temporal | RD | -3.36 | 1.85 | -1.82 | .071 | .406 |
|  |  | Vocabulary Subgroup | 0.03 | 0.18 | 0.19 | .846 | .998 |
|  |  | RD x Group | 3.29 | 1.94 | 1.70 | .093 | .341 |
| All analyses additionally controlled for Time 1 age, Time 1 EOWPVT raw score, sex assigned at birth, average head motion and scanner system  RD, Radial Diffusivity; Arcuate Fasciculus; ILF, Inferior Longitudinal Fasciculus; CST, Corticospinal Tract; UF, uncinate fasciculus; EOWPVT, Expressive One Word Picture Vocabulary Test | | | | | | | |

**Table S13. EOWPVT change rate predicted by white matter FA and behavior in autistic with smaller spoken vocabularies at Time 1**

| ROI | Predictor | Sum Sq | DF | F | p | Percent Variance |
| --- | --- | --- | --- | --- | --- | --- |
| AF Left | Intercept | 0.82 | 1 | 3.55 | .07 | 5.42 |
| Central | FA (Time 1) | 1.12 | 1 | 4.87 | .03 | 7.42 |
|  | Joint Attention (Time 1) | 4.38 | 1 | 18.97 | <.001 | 28.92 |
|  | Fine Motor Skills (Time 1) | 0.78 | 1 | 3.37 | .08 | 5.13 |
|  | EOWPVT raw score (Time 1) | 0.40 | 1 | 1.71 | .20 | 2.61 |
|  | Age (Time 1) | 0.17 | 1 | 0.72 | .40 | 1.10 |
|  | Sex (Time 1) | 0.09 | 1 | 0.39 | .54 | 0.60 |
| ILF Left | Intercept | 0.84 | 1 | 4.94 | .03 | 5.43 |
| Occipital | FA (Time 1) | 3.10 | 1 | 18.30 | <.001 | 20.11 |
|  | Joint Attention (Time 1) | 4.31 | 1 | 25.48 | .001 | 28.00 |
|  | Fine Motor Skills (Time 1) | 0.79 | 1 | 4.67 | .04 | 5.14 |
|  | EOWPVT raw score (Time 1) | 0.18 | 1 | 1.08 | .31 | 1.19 |
|  | Age (Time 1) | 0.36 | 1 | 2.13 | .15 | 2.34 |
|  | Sex (Time 1) | 0.40 | 1 | 2.38 | .13 | 2.61 |
| CST Left | Intercept | 0.79 | 1 | 3.44 | .07 | 5.03 |
| Inferior | FA (Time 1) | 1.12 | 1 | 4.85 | .03 | 7.09 |
|  | Joint Attention (Time 1) | 4.98 | 1 | 21.54 | <.001 | 31.47 |
|  | Fine Motor Skills (Time 1) | 0.91 | 1 | 3.94 | .06 | 5.75 |
|  | EOWPVT raw score (Time 1) | 0.39 | 1 | 1.69 | .20 | 2.47 |
|  | Age (Time 1) | 0.17 | 1 | 0.74 | .39 | 1.09 |
|  | Sex (Time 1) | 0.05 | 1 | 0.23 | .63 | 0.34 |
| Diffusion parameters were residualized for average head motion and scanner system  FA, Fractional Anisotropy; AF, Arcuate Fasciculus; ILF, Inferior Longitudinal Fasciculus; CST, corticospinal tract; EOWPVT, Expressive One Word Picture Vocabulary Test | | | | | | |

**Table S14. Summary of exploratory mediation analyses**

| Predictors | Mediator | Outcome | Total Effect | Direct Effect | Indirect Effect (95% CI Bootstrap) |
| --- | --- | --- | --- | --- | --- |
| FM  JA | AF Left Central | EOWPVT Change Rate | -0.172 0.036  0.033 0.027 | -0.161(0.039)  0.038(0.025) | -0.011 (-0.047,0.008)  -0.005 (-0.027, 0.019) |
| FM  JA | ILF Left Occipital | EOWPVT Change Rate | 0.033(0.027)  0.033(0.027) | -0.170(0.039)  0.033(0.027) | -0.002(-0.043,0.054)  0.000(-0.031,0.033) |
| FM  JA | CST Left Inferior | EOWPVT Change Rate | -0.172(0.036)  0.033(0.028) | -0.172(0.040)  0.034(0.029) | 0.000(-0.037,0.013)  -0.001(-0.029,0.011) |
| FM, Fine Motor; JA, Joint Attention; AF, Arcuate Fasciculus; ILF, Inferior Longitudinal Fasciculus; CST, Corticospinal Tract; EOWPVT, Expressive One Word Picture Vocabulary Test | | | | | |
